# People Who Identify with All of Humanity Make Less of a Moral Distinction Between Close and Distant People

**DOI:** 10.3390/bs16091541

**Published:** 2026-09-01

**Authors:** Siqi Zhao, Ziyan Wang, Huarong Liu, Qilong Li, Yumeng Sun, Kaiping Peng, Xiaomeng Hu

**Affiliations:** 1 Department of Psychology, Renmin University of China, Beijing 100872, China; 2022102281@ruc.edu.cn (S.Z.); wzy26@ruc.edu.cn (Z.W.); 2023101969@ruc.edu.cn (H.L.); liqilong20@ruc.edu.cn (Q.L.); 2025103903@ruc.edu.cn (Y.S.); 2 Department of Psychological and Cognitive Sciences, Tsinghua University, Beijing 100084, China

**Keywords:** identification with all humanity, moral differentiation mode, impartial beneficence, empathy

## Abstract

In the era of promoting the concept of a “Community with a Shared Future for Mankind,” understanding the psychological mechanisms underlying relational morality in Chinese culture holds important theoretical and practical value, particularly given its hierarchical structure of moral obligations. Across four studies, this research innovatively proposes a calculation method for moral differential mode to deeply explore how identification with all humanity (IWAH) influences Chinese people’s moral differential mode. Study 1 used questionnaires to preliminarily explore the differential characteristics of Chinese people’s moral circles and found that IWAH significantly reduced moral differential mode. Study 2 verified these findings and explored the mediating role of impartial beneficence, initially constructing a mediation model. Study 3 employed experimental methods to manipulate IWAH through priming paradigms, further verifying its causal effect on moral differential mode and the mediating role of impartial beneficence. Study 4 introduced empathy as a moderating variable and found that personal distress and perspective taking moderated the effect of IWAH on moral differentiation mod. Together, these findings provide novel empirical insights into the dynamics of cultural change and social adaptation. They demonstrate how promoting global identification can expand traditional cultural norms, offering psychological pathways to foster inclusive social attitudes crucial for global cooperation and sustainable human development.

## 1. Introduction

In an era where human sustainable development is threatened by global challenges such as climate change and public health crises, addressing these issues requires unprecedented global cooperation and a profound cultural shift in social attitudes. Identification with all humanity (IWAH) refers to a psychological disposition in which individuals perceive all human beings as members of their ingroup and demonstrate active concern for the entire human family (McFarland & Brown, 2008; McFarland et al., 2012). This construct offers a theoretical foundation for understanding individual differences in endorsement of the “community with a shared future for mankind” and examining its psychological and behavioral implications. Prior research suggests that IWAH is associated with a range of beneficial psychological and behavioral outcomes, including enhanced cross-group interactions (Hamer et al., 2017, 2018; Reese & Kohlmann, 2015), greater willingness to engage in pro-environmental behaviors (Aydın et al., 2022; Pong & Tam, 2023), and increased motivation to respond to public health emergencies (Deng, 2021; Sparkman, 2023). Nevertheless, current research on IWAH has primarily concentrated on its facilitative effects on specific moral actions, with limited attention to how it shapes individuals’ underlying moral psychological structures.

Concurrently, moral psychology has undergone substantial shifts, notably the expansion of the moral circle envisioned by philosophers and psychologists (Singer, 1981; Crimston et al., 2018). From ancient Rome—where spectacles such as slave fights were widely accepted—to modern societies where animals, plants, and other marginalized entities are increasingly being accorded moral consideration and legal protection (Bloom, 2010; Crimston et al., 2016; Pinker, 2011), this phenomenon has been extensively examined by Western scholars from both ethical and psychological perspectives. Nonetheless, moral circle theory remains rooted in Western cultural traditions and predominantly emphasizes the broadening of moral boundaries (Crimston et al., 2016; Yu & Xu, 2018). When examining Chinese moral psychology, one must account for the distinctive features of local culture and social organization. Western societies often operate on an organizational mode analogous to a bundle of straws, characterized by clear, well-defined group boundaries (Fei, 2019). In contrast, Chinese society is organized according to a differential mode of association (chaxugeju). Fei metaphorically described this structure as being like a stone thrown into a lake: the self is at the center, and social relationships form concentric ripples that extend outward, representing decreasing degrees of relational and emotional intimacy. The structural differences between these two social systems lead to distinct moral conceptions. Informed by Confucian ethics, which emphasizes that ‘relatives and friends have different degrees of closeness and intimacy,’ Chinese moral reasoning often involves differential moral concern based on relational proximity and one’s position within these concentric circles—a pattern referred to as “moral differential mode.” Amid global change, China promotes the ethical ideal of “advancing together with others toward benevolence” (Zeng, 2020). However, how contemporary Chinese individuals reconcile the traditional notion of “differential reality” with the global ideal of a unified moral community warrants further empirical investigation and represents a vital process of cultural adaptation.

Building upon this context, the present research seeks to systematically examine the influence of IWAH on moral differential mode and its underlying psychological mechanisms. Specifically, drawing on social identity theory, the common ingroup identity model, and the utilitarian two-dimensional framework, the study adopts both questionnaire-based and experimental methodologies to assess the mediating role of impartial beneficence in the relationship between IWAH and moral differential mode. Furthermore, it investigates whether four dimensions of empathy—personal distress, empathic concern, perspective taking, and fantasy—moderate this relationship. Through this multi-method approach, the present study aims to deepen understanding of the psychological benefits associated with IWAH, develop a culturally sensitive measure of moral differential mode tailored to the Chinese context, and contribute to the broader framework of cross-cultural moral psychology.

## 2. Literature Review

### 2.1. The Impact of IWAH on Moral Differential Mode

Social identity theory suggests that individuals’ identification with an ingroup enhances their perception of ingroup members and prioritizes the group’s collective interests. Furthermore, individuals are often motivated to perceive the protection of ingroup interests as a moral obligation, adjusting their psychological orientations and behaviors accordingly (Turner, 1999). When individuals come to perceive all humanity as their ingroup, this form of global identification fosters an awareness of a shared human destiny and a corresponding moral obligation to promote the welfare of all people, thereby encouraging an orientation toward impartial beneficence. This, in turn, encourages individuals to adopt a more equitable moral stance that aims to maximize the well-being of humanity at large, rather than privileging close others. Empirical research has demonstrated that individuals with strong global identification show heightened concern for global issues, such as eradicating hunger (McFarland et al., 2012) and addressing inequality (McFarland & Hornsby, 2015), indicating a universalistic moral orientation. Global identity has also been linked to increased willingness to donate to victims of international disasters (McFarland et al., 2012) and to support for providing aid and shelter to refugees (Bassett & Cleveland, 2019). These findings suggest that moral concern for outgroups is a critical antecedent of prosocial attitudes and behaviors directed beyond one’s immediate social group. Notably, IWAH does not appear to diminish moral concern for primary ingroups. Studies have found that individuals high in global identity often simultaneously endorse strong national and community identification (Bassett & Cleveland, 2019; Bayram, 2018; Hamer & Gutowski, 2009) and show heightened generosity toward ingroup members (Grimalda et al., 2018). This pattern likely extends to the Chinese context, where traditional and modern value systems coexist in a non-antagonistic manner (Huang et al., 2018). For instance, filial piety and familial obligations remain central to social functioning in contemporary Chinese society (Yang, 2004; Xu & Hamamura, 2014). As such, IWAH is unlikely to diminish moral concern for proximal relational groups. Rather, it may expand moral concern to more peripheral entities without weakening commitment to core relationships, thereby reducing moral differential mode.

**H1.**  *Identification with all humanity negatively predicts moral differential mode.*

### 2.2. The Mediating Role of Impartial Beneficence Between IWAH and Moral Differential Mode

Social identity theory posits that identification with an ingroup enhances individuals’ perception of ingroup members and promotes alignment with group interests (Turner, 1999). This identification often leads individuals to perceive the protection of those interests as a moral obligation, guiding their cognitive and behavioral tendencies accordingly. When individuals conceptualize all humanity as a single ingroup, this universal identification fosters a sense of shared human destiny and a corresponding obligation to promote global well-being, thereby reinforcing their orientation toward impartial beneficence. As a result, individuals adopt a more equitable moral stance aimed at maximizing the welfare of all people, rather than prioritizing close others.

Empirical research supports this assertion. For example, individuals with high levels of global identification demonstrate greater concern for global issues such as alleviating hunger (McFarland et al., 2012) and addressing economic inequality (McFarland & Hornsby, 2015), indicating a universalistic moral stance. Moreover, social identity has been shown to significantly influence moral judgment (Abel & Hornsey, 2010). IWAH has been found to positively predict impartial beneficence and to negatively predict instrumental harm (Kahane et al., 2018), suggesting that globally identified individuals both prioritize collective human welfare and reject the use of harm as a means to achieve moral ends.

Importantly, impartial concern influences the structure of moral concern itself. This architecture is malleable and subject to modulation by cognitive processes such as abstraction and perspective-taking (Crimston et al., 2018; Laham, 2009). Endorsing impartial beneficence involves advocating for the greater good in an unbiased and egalitarian manner—without privileging others based on relational proximity or familiarity. Accordingly, individuals who endorse impartiality tend to extend more uniform moral consideration across diverse targets, ultimately reducing moral differential mode.

**H2.**  *Impartial beneficence partially mediate the relationship between IWAH and moral differential mode.*

### 2.3. The Moderating Role of Empathy Between IWAH and Moral Differential Mode

Batson et al. (1987) noted that when individuals face others’ misfortunes, they experience two primary emotional responses: empathetic concern and personal distress. The former manifests as warm, other-oriented compassion, while the latter reflects self-centered discomfort and anxiety, each leading to different motivations and outcomes. According to the empathy–altruism hypothesis, empathy-based concern is a core motivator of altruistic behavior, prompting individuals to focus on others’ well-being and inspiring prosocial actions. In contrast, personal distress leads individuals to adopt avoidance strategies to alleviate their own discomfort, such as avoiding contact with those in distress (Batson, 1991). Personal distress only promotes helping behavior when individuals perceive altruistic action as the most effective way to reduce their own discomfort (Batson, 1991). Empirical research in moral psychology has further revealed that empathy and personal distress exert distinct effects. For example, empathy facilitates altruistic responses and mature prosocial moral reasoning, whereas personal distress reinforces moral disengagement mechanisms (Paciello et al., 2013). In the context of the relationship between IWAH and moral differential mode, these two emotional responses function as critical boundary conditions. Individuals with high empathic concern are more likely to genuinely care for the well-being of diverse entities, including marginalized groups, thereby strengthening the negative predictive effect of IWAH on the moral differential mode. Conversely, when individuals with high personal distress are confronted with the hardships of peripheral entities (such as stigmatized groups), they may adopt avoidance strategies—intentionally ignoring these marginalized entities to alleviate their own anxiety and discomfort. Consequently, this avoidance tendency attenuates the inclusive effect of IWAH, preventing it from effectively reducing the moral differential mode.

**H3.**  *Personal distress moderates the relationship between IWAH and moral differential mode. Specifically, individuals with lower levels of personal distress experience a relatively stronger reduction in moral differential mode associated with IWAH.*

**H4.**  *Empathic concern moderates the relationship between IWAH and moral differential mode. Specifically, individuals with higher levels of empathic concern experience a relatively stronger reduction in moral differential mode.*

Perspective-taking, defined as the cognitive process of adopting another’s viewpoint, reduces intergroup stereotypes and fosters intergroup understanding (Galinsky & Moskowitz, 2000). However, it may also produce adverse effects. Vorauer et al. (2009) found that individuals with low levels of prejudice may become complacent during intergroup interactions, assuming they will be positively perceived by outgroup members and thereby showing reduced attentiveness. In competitive contexts, perspective-taking may lead individuals to overestimate others’ self-interested motives, resulting in more reactive egoism (Epley et al., 2006). In cultures with hierarchical moral structures, individuals with high levels of perspective-taking may assume that others also accept moral stratification, which may reinforce the existing moral order. Hortensius et al. (2016) observed that in emergency situations, high perspective-taking individuals exhibit stronger bystander effects due to greater cognitive load, which delays decision-making. When encountering moral dilemmas, such individuals may perceive complex relational demands and the relativity of moral obligations—for instance, understanding the needs of family members in the moral inner circle alongside those of marginalized groups—which may lead to sustaining differentiated moral concern and weaken the equalizing impact of IWAH. In a culture deeply rooted in the differential mode of association, individuals with high perspective-taking might also assume that others recognize and accept the legitimacy of moral stratification. By fully comprehending the culturally “justified” differences in moral standing between core family members and peripheral outgroups, they may choose to maintain, rather than challenge, the existing moral hierarchy. Thus, high perspective-taking may weaken the equalizing impact of IWAH on the moral differential mode.

Although research on fantasy and moral concern remains limited, Pinker (2011) argued that moral expansion can occur through indirect means such as engaging with the literature. Individuals with greater imaginative capacity may more vividly simulate and empathize with the experiences of marginalized groups, thereby extending moral concern and reducing hierarchical distinctions in moral judgment. Therefore, heightened levels of fantasy may enhance the capacity of IWAH to break down traditional moral boundaries, amplifying its negative predictive effect on the moral differential mode.

**H5.**  *Perspective-taking moderates the relationship between IWAH and moral differential mode. Specifically, for individuals with low levels of perspective-taking, the negative predictive effect of IWAH on moral differential mode is relatively stronger.*

**H6.**  *Fantasy moderates the relationship between IWAH and moral differential mode. Specifically, for individuals with high levels of fantasy, the reduction in moral differential mode due to IWAH is relatively stronger.*

## 3. Study 1: The Impact of IWAH on Moral Differential Mode

### 3.1. Method

Study 1 employs a questionnaire-based design to measure participants’ levels of IWAH, moral differential mode, and demographic variables, with the goal of constructing an initial model of their interrelationships.

#### 3.1.1. Procedure and Participants

A total of 301 valid responses were collected, including 173 males and 128 females, with a mean age of 25.27 years (*SD* = 6.26). Participants were recruited through an online platform, and the survey was administered using the Chinese survey platform “Wenjuanxing.” To ensure data quality, attention check items (e.g., “Please select option X for this question”) were included.

#### 3.1.2. Measures

This study employed the Chinese version of the Identification With All Humanity Scale (IWAH; McFarland et al., 2012), adapted by S. X. Chen et al. (2023) (see Appendix A). The scale consists of 9 items, with a sample item being: “How close do you feel to each of the following groups?—People in my community, People in my country, People anywhere in the world.” For each item, participants rated three distinct target groups separately: “people in my community,” “people in my country,” and “people anywhere in the world.” Responses were recorded on a 6-point Likert scale ranging from 1 (not at all) to 6 (very much). The mean score for the “people anywhere in the world” subscale was calculated to represent participants’ level of IWAH, with higher scores indicating stronger global identification. In the present study, the Cronbach’s *α* coefficient for the IWAH scale was 0.902.

The study employed an adapted version of the Moral Expansiveness Scale (MES; Crimston et al., 2016; see Appendix B). Participants first read an instructional guide outlining the definition of each moral boundary, such as: “Inner Circle of Moral Concern: These entities deserve the highest level of moral concern and standing. You have a moral obligation to ensure their welfare and feel a sense of personal responsibility for their treatment.” They were then asked to assign 18 human-related entities into one of the four concentric circles, using a 4-point scoring system (0 = Outside the Moral Boundary, 3 = Inner Circle of Moral Concern). To account for cultural differences, several items from the original scale were adaptively adjusted; for instance, “member of opposing political party” was replaced with “member of a different organization.” Following the methodology of Rottman et al. (2021), exploratory factor analysis was conducted to determine the specific entities belonging to each circle to calculate the average moral concern score for each layer. The difference in mean scores between the entities in the inner circle and those on the fringes was computed to represent the moral differential mode. In the present study, the Cronbach’s *α* coefficient for the Moral Expansiveness Scale was 0.89.

Demographic Variables. Demographic data such as gender and age were also collected.

### 3.2. Results

#### 3.2.1. Common Method Bias Test

As the data were collected through self-report questionnaires, exploratory factor analysis was used to assess potential common method bias (Zhou & Long, 2004). Harman’s single-factor test revealed that the first factor accounted for 28.20% of the variance, which is below the recommended threshold of 40%. Therefore, no substantial common method bias was detected.

#### 3.2.2. Exploratory Factor Analysis

First, the Kaiser–Meyer–Olkin (KMO) measure and Bartlett’s test of sphericity were conducted on the moral expansiveness scale scores. The results indicated that KMO = 0.901, *χ*^2^ = 2307.791, *df* = 153, *p* < 0.001, suggesting the data were suitable for factor analysis. We then conducted an exploratory factor analysis using principal axis factoring with promax rotation. Principal axis factoring was selected because the aim was to estimate common latent factors and because it is less dependent on multivariate normality than maximum likelihood extraction. Promax, an oblique rotation, was used because moral concern across different categories of targets was theoretically expected to be correlated. Consistent with the four-layer organization of moral expansiveness, a four-factor solution was examined.

The four-factor solution accounted for approximately 52% of the item variance and showed acceptable fit, TLI = 0.924, RMSEA = 0.059, 90% CI [0.047, 0.072], and RMSR = 0.030. The pattern matrix was broadly interpretable as Outer Circle of Moral Concern, Fringes of Moral Concern, Outside the Moral Boundary, and Inner Circle of Moral Concern. The correlations among the extracted factors ranged from −0.09 to 0.68; notably, the Outer Circle and Fringes factors were correlated at 0.68, supporting the use of an oblique rather than orthogonal rotation. Most targets had their largest loading on the theoretically interpretable factor. Refugee and member of a different organization showed cross-loadings on the Outer Circle and Fringes factors, with their larger loadings occurring on the Fringes factor. The complete pattern matrix, including all cross-loadings, is reported in Table 1.

Following Rottman et al. (2021), the average score for each factor was calculated to represent individuals’ moral concern toward different circles. The average score difference between the inner circle and fringes of moral concern was computed to represent moral differential mode, reflecting the degree of variation in moral concern across different social entities.

#### 3.2.3. Correlation Analysis

Descriptive statistics are presented in Table 2. A significant negative correlation was observed between IWAH and moral differential mode (*r* = −0.37, *p* < 0.001). IWAH did not show a significant correlation with moral concern outside the moral boundary (*ps* > 0.450), but was significantly positively correlated with moral concern in the outer circle (*r* = 0.33, *p* < 0.001) and the fringes of moral concern (*r* = 0.38, *p* < 0.001).

Moral differential mode exhibited a significant positive correlation with the inner circle of moral concern (*r* = 0.19, *p* = 0.001), but was significantly negatively correlated with the outer circle (*r* = −0.53, *p* < 0.001), the fringes of moral concern (*r* = −0.89, *p* < 0.001), and outside the moral boundary (*r* = −0.14, *p* = 0.012). At different levels of moral concern, significant positive correlations were found between the inner and outer circles (*r* = 0.38, *p* < 0.001), the inner circle and the fringes of moral concern (*r* = 0.29, *p* < 0.001), and the outer circle and the fringes of moral concern (*r* = 0.69, *p* < 0.001).

No significant relationships were observed between age and IWAH or moral differential mode (*ps* > 0.280). However, age showed a significant positive correlation with moral concern in the outer circle (*r* = 0.19, *p* = 0.001).

#### 3.2.4. Regression Analysis

A regression model was constructed with IWAH as the independent variable and moral differential mode as the dependent variable (*R*^2^ = 0.14, *F*(1, 299) = 47.08, *p* < 0.001). The results showed that IWAH was a significant negative predictor of moral differential mode (*B* = −0.26, *p* < 0.001). After controlling for age and gender, with gender treated as a categorical covariate, IWAH remained a significant negative predictor of moral differential mode (*R*^2^ = 0.14, *F*(3, 297) = 15.96, *p* < 0.001), indicating that the negative effect of IWAH on moral differential mode remained robust after accounting for covariates (*B* = −0.26, *p* < 0.001). Detailed results are presented in Table 3.

Another regression model was constructed with IWAH as the independent variable and moral concern as the dependent variable (*R*^2^ = 0.002, *F*(1, 299) = 0.57, *p* = 0.450). The results indicated that IWAH was not a significant predictor of moral concern (*B* = 0.02, *p* = 0.450). After controlling for age and gender, with gender treated as a categorical covariate, the multiple regression model continued to be nonsignificant (*R*^2^ = 0.02, *F*(3, 297) = 1.69, *p* = 0.169). Detailed results are shown in Table 4.

A third regression model was constructed with IWAH as the independent variable and the fringes of moral concern as the dependent variable (*R*^2^ = 0.14, *F*(1, 299) = 50.43, *p* < 0.001). The results showed that IWAH was a significant positive predictor of the fringes of moral concern (*B* = 0.28, *p* < 0.001). After controlling for age and gender, with gender treated as a categorical covariate, the multiple regression model remained significant (*R*^2^ = 0.15, *F*(3, 297) = 16.80, *p* < 0.001), indicating that the positive effect of IWAH on the fringes of moral concern remained robust (*B* = 0.28, *p* < 0.001). Specific results are presented in Table 5.

### 3.3. Discussion

Study 1 preliminarily validated the differential structure of the Chinese moral circle and found that IWAH can significantly negatively predict moral differentiation.

The factor analysis results of Study 1 indicate that the moral concern of Chinese participants is similar to that of American participants (Crimston et al., 2016), exhibiting a four-tiered structure that decreases from the inner to the outer layers: family members and close friends occupy the core position of moral concern; in-groups (e.g., Chinese citizen) and respected groups (e.g., charity workers) are in the outer circles of moral concern; stigmatized groups (e.g., individual with a mental illness) and out-groups (e.g., foreign citizen ) are at the fringes of moral concern; and evildoers are outside the moral boundary.

Consistent with H1, IWAH significantly negatively predicts moral differential mode, meaning that when individuals tend to view all humanity as a unified whole, their moral care differences toward different entities significantly decrease. Additionally, IWAH is significantly positively correlated with moral care scores for outer circle and fringes targets, but not significantly correlated with scores for inner circle and outside the moral boundary entities. This suggests that IWAH does not weaken moral concern for close groups but enhances moral concern for peripheral groups, thereby reducing moral differential mode.

## 4. Study 2: Identification with All Humanity on Reducing Moral Differential Mode: The Mediating Role of Impartial Beneficence

### 4.1. Method

Study 2 aims to replicate and extend the findings of Study 1 by re-examining the hierarchical nature of the moral circle and the association between IWAH and moral differentiation, while investigating the mediating role of impartial beneficence. A questionnaire-based approach is used to measure participants’ IWAH, impartial beneficence, and moral differential mode, as well as demographic characteristics and instrumental harm (as control variables), in order to test a mediation model in which IWAH reduces moral differential mode via impartial beneficence.

#### 4.1.1. Procedure and Participants

A total of 303 valid responses were obtained, consisting of 143 males and 160 females, with a mean age of 23.50 years (*SD* = 4.27). Participants were recruited via an online platform, and the survey was administered through the Chinese online survey tool “Wenjuanxing.” To ensure data quality, attention-check items (e.g., “Please select option X for this question”) were included in the questionnaire.

#### 4.1.2. Measures

IWAH and moral differential mode were measured using the same scales as in Study 1.

The Chinese version of the Oxford Utilitarianism Scale (OUS; Kahane et al., 2018) was used, adapted by M. Chen et al. (2021). (see Appendix C). This scale comprises 9 items divided into two distinct subscales: Impartial Beneficence and Instrumental Harm. The Impartial Beneficence subscale consists of 5 items (e.g., “From a moral point of view, we should feel obliged to give one of our kidneys to a person with kidney failure since we don’t need two kidneys to survive, but really only one to be healthy”). The Instrumental Harm subscale consists of 4 items (e.g., “It is morally right to harm an innocent person if harming them is a necessary means to helping several other innocent people”). Participants rated their agreement with each statement on a 6-point Likert scale ranging from 1 (strongly disagree) to 6 (strongly agree). Mean scores were calculated for each subscale, with higher scores reflecting greater tendencies toward impartial beneficence or instrumental harm. In the present study, the Cronbach’s *α* coefficient for the overall scale was 0.890, while the reliabilities for the Impartial Beneficence and Instrumental Harm subscales were 0.79 and 0.86, respectively.

Demographic Variables. Demographic information, including gender and age, was also collected.

### 4.2. Results

#### 4.2.1. Statistical Analysis

Following the exploratory analysis in Study 1, the four-factor target structure was evaluated using confirmatory factor analysis in Studies 2–4. The same Study 1 target-to-factor assignments were specified in each sample, and no target was reassigned on the basis of the results of an individual study. Models were estimated using robust maximum likelihood (MLR), which provides robust standard errors and scaled test statistics under nonnormality. Model fit was evaluated using the robust comparative fit index (CFI), robust Tucker–Lewis index (TLI), robust root mean square error of approximation (RMSEA) with its 90% confidence interval, and the standardized root mean square residual (SRMR). Because fit indices represent different aspects of fit, they were considered jointly rather than using a single index as a definitive criterion.

Data analysis was conducted using SPSS 26.0. The primary analyses consisted of tests for common method bias, confirmatory factor analysis, descriptive statistics, correlation analysis, and mediation model testing.

#### 4.2.2. Common Method Bias Test

As the data were collected through self-report questionnaires, an exploratory factor analysis was conducted to examine potential common method bias (Zhou & Long, 2004). According to Harman’s single-factor test, the first factor accounted for 19.55% of the total variance, which is below the recommended threshold of 40%. Therefore, no serious common method bias was present in the dataset.

#### 4.2.3. Confirmatory Factor Analysis

A confirmatory factor analysis tested the four-factor structure obtained in Study 1. The model provided acceptable overall fit, scaled *χ*^2^ (129) = 193.50, *p* < 0.001, robust CFI = 0.947, robust TLI = 0.937, robust RMSEA = 0.041, 90% CI [0.026, 0.054], and SRMR = 0.049. The standardized factor loadings ranged from 0.51 to 0.74 and were all statistically significant. These results provided support for applying the Study 1 target grouping in Study 2.

#### 4.2.4. Correlation Analysis

The results of the descriptive statistical analysis are presented in Table 6. Additional demographic characteristics of the participants (i.e., education, income, and socioeconomic status) are detailed in Table S1 of the Supplementary Materials. IWAH was significantly positively associated with impartial beneficence (*r* = 0.21, *p* < 0.001) and significantly negatively associated with moral differential mode (*r* = −0.27, *p* < 0.001). Impartial beneficence was significantly negatively associated with moral differential mode (*r* = −0.22, *p* < 0.001) and significantly positively associated with instrumental harm (*r* = 0.73, *p* < 0.001). The control variable instrumental harm was not significantly associated with either IWAH or moral differential mode (*ps* > 0.069). In addition, age was significantly positively associated with both instrumental harm (*r* = 0.16, *p* = 0.005) and impartial beneficence (*r* = 0.17, *p* = 0.003).

#### 4.2.5. Mediating Model Testing

Using Model 4 from the PROCESS macro in SPSS 26.0 (Hayes, 2013), a mediation model was constructed with impartial beneficence as the mediator between IWAH and moral differential mode. IWAH was specified as the independent variable, moral differential mode as the dependent variable, and demographic variables along with instrumental harm were included as control variables.

**Figure 1:**
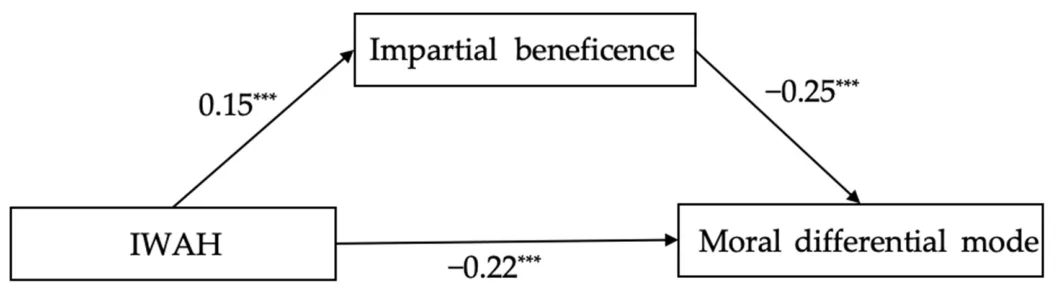
Mediation Model in Study 2. *** *p* < 0.001.

The path coefficients are presented in Figure 1 and detailed in Table 7 IWAH continued to significantly and positively predict impartial beneficence (*B* = 0.17, *SE* = 0.04, *β* = 0.15, *t* = 3.88, *p* < 0.001), and significantly and negatively predicted moral differential mode (*B* = −0.12, *SE* = 0.03, *β* = −0.22, *t* = −3.94, *p* < 0.001). Impartial beneficence significantly negatively predicted moral differential mode (*B* = −0.11, *SE* = 0.04, *β* = −0.25, *t* = −3.02, *p* = 0.003). The mediating effect of impartial beneficence was further tested via a bootstrap analysis with 10,000 samples. The 95% bootstrap confidence interval ranged from −0.08 to −0.01, again excluding zero, confirming a significant mediating effect. Additionally, the effects of gender and age were not statistically significant (*p*s > 0.143). Instrumental harm significantly predicted impartial beneficence (*B* = 0.62, *SE* = 0.03, *β* = 0.71, *t* = 17.85, *p* < 0.001), but did not significantly predict moral differential mode (*p* = 0.269). The direct and indirect effects are summarized in Table 8.

### 4.3. Discussion

Study 2 further validated the distinctive hierarchical structure of the Chinese moral circle and demonstrated that IWAH significantly and negatively predicts moral differentiation. Additionally, the analysis revealed that tendencies toward impartial beneficence partially mediated the relationship between IWAH and moral differentiation.

Consistent with H2, the mediation analysis indicated that IWAH reduces moral differentiation by enhancing individuals’ tendencies toward impartial beneficence. When individuals identify with and express concern for all humanity, they are more likely to adopt an impartial beneficence orientation, prioritizing the maximization of overall well-being rather than the exclusive interests of proximal groups. This, in turn, narrows the moral concern gap across different social circles.

This study also found that IWAH was significantly positively correlated with impartial beneficence but showed no significant correlation with instrumental harm. This suggests that IWAH does not promote the instrumental harm mindset of “sacrificing the few for the greater good,” but instead reinforces endorsement of impartial beneficence. This distinction deepens our understanding of the moral implications of IWAH. Furthermore, moral differential mode was significantly negatively correlated with impartial beneficence but unrelated to instrumental harm, reaffirming that impartial beneficence and instrumental harm represent two relatively independent dimensions and supporting the two-dimensional model of utilitarianism.

Moreover, impartial beneficence and instrumental harm were positively correlated within the Chinese sample, a pattern consistent with previous findings from Chinese participants (M. Chen et al., 2021) and samples of Western philosophers (Kahane et al., 2018). This suggests that, although these are distinct dimensions, they may share an underlying theoretical basis. Finally, the finding that age was positively correlated with both impartial beneficence and instrumental harm contradicts prior findings by Kahane et al. (2018). As the present sample was relatively young, future studies should further examine these associations using more representative samples.

## 5. Study 3: The Causal Relationship Between Identification with All Humanity and Moral Differential Mode, and the Mediating Role of Impartial Beneficence

### 5.1. Method

Study 3 aims to further investigate the causal relationship between IWAH and moral differential mode using an experimental design and to test the mediating role of impartial beneficence. Specifically, Study 3 will employ an experimental paradigm to test whether the activation of IWAH enhances individuals’ tendencies toward impartial beneficence, thereby reducing their moral differential mode.

#### 5.1.1. Participants

A total of 151 valid responses were collected, including 59 male and 92 female participants, with a mean age of 23.40 years (*SD* = 3.58). Participants were recruited through an online recruitment platform, and data collection was carried out using the Chinese survey platform “Wenjuanxing.” Participants were randomly assigned to one of two conditions: 76 in the priming group and 75 in the control group.

#### 5.1.2. Procedure

This study employed a single-factor between-subjects experimental design based on the paradigm used by Sparkman et al. (2022). Participants first received detailed information about the study’s purpose and the measures taken to ensure data confidentiality, and then provided informed consent. After reading the experimental instructions, they were randomly assigned to one of two experimental conditions: the priming group or the control group. Following the reading of the materials, participants responded to comprehension questions related to the materials. They then completed the Identification With All Humanity Scale (IWAH; McFarland et al., 2012), which was used as the manipulation check, followed by the Impartial Beneficence Subscale and the Moral Expansiveness Scale. Finally, participants provided demographic information and completed the Instrumental Harm Subscale.

#### 5.1.3. Materials of Priming Identification with All Humanity

Based on previous research (Reese & Kohlmann, 2015; Sparkman et al., 2022), this experiment adopted a pictorial and textual priming paradigm, comprising two experimental conditions: an identification-with-all-humanity priming group and a blank control group. The manipulation materials for identification with all humanity were adapted from the study by Sparkman et al. (2022).

Participants in the priming group viewed an image of the Earth and read a text designed to enhance the priming effect (see Appendix D). After carefully viewing the image and reading the text, they were required to answer the following questions: “Do you agree with the viewpoints presented in the text? How would you understand and uphold the human family?”

Participants in the control group viewed a blank image and read a descriptive text about the image (see Appendix D). After carefully viewing the image and reading the text, they were required to answer the following questions: “Do you agree with the viewpoints presented in the text? How would you understand and use the color white?”

#### 5.1.4. Measures

The Chinese versions of the IWAH Scale, the Moral Expansiveness Scale, and the Oxford Utilitarianism Scale were used to measure identification with all humanity, moral circles, and impartial beneficence, respectively. All scales were identical to those employed in Study 2. Demographic Variables. Participants also reported demographic information, including gender and age.

### 5.2. Results

#### 5.2.1. Common Method Bias Test

Since the data for this study were collected using questionnaires, exploratory factor analysis was used to test for possible common method bias (Zhou & Long, 2004). Using Harman’s single-factor test, the first common factor explained 20.70% of the variance, which is less than 40%. Therefore, there is no serious common method bias in the data for this study.

#### 5.2.2. Confirmatory Factor Analysis

The same four-factor model was also examined in the experimental sample. Model fit was mixed, scaled *χ*^2^ (129) = 235.23, *p* < 0.001, robust CFI = 0.866, robust TLI = 0.841, robust RMSEA = 0.066, 90% CI [0.049, 0.083], and SRMR = 0.070. Although the absolute fit indices were within commonly used acceptable ranges, the incremental fit indices were below conventional benchmarks. The weaker fit was concentrated particularly in the two-indicator Inner Circle factor. Given the smaller sample size of Study 3 and its primary purpose as an experimental test rather than a scale-validation study, these results were treated as qualified rather than conclusive support for the four-factor measurement structure. To preserve comparability, the Study 1 scoring rule was retained without data-driven reassignment of targets.

#### 5.2.3. Correlation Analysis

The results of the descriptive statistical analysis are presented in Table 9. Impartial beneficence was significantly negatively correlated with moral differential mode (*r* = −0.32, *p* < 0.001) and positively correlated with instrumental harm (*r* = 0.54, *p* < 0.001). The control variable, instrumental harm, was not significantly correlated moral differential mode (*ps* > 0.564). However, a marginally significant correlation was observed between age and moral differential mode (*r* = 0.16, *p* = 0.050).

#### 5.2.4. Manipulation Check

An independent-samples *t*-test was conducted to examine whether the priming manipulation increased participants’ identification with all humanity. The manipulation check was based on the IWAH items referring to “people all over the world” (Cronbach’s *α* = 0.916). Levene’s test indicated that the homogeneity of variance assumption was met, *F* = 0.54, *p* = 0.464. Participants in the priming condition reported significantly higher IWAH scores (*M* = 4.34, *SD* = 0.81) than those in the control condition (*M* = 3.37, *SD* = 0.73), *t*(149) = 7.78, *p* < 0.001, Cohen’s *d* = 1.27. These results indicated that the priming manipulation successfully increased IWAH.

#### 5.2.5. Impartial Beneficence

An independent-samples *t*-test was conducted to compare impartial beneficence scores between the priming and control conditions. Levene’s test indicated that the homogeneity of variance assumption was met, *F* = 2.82, *p* = 0.095. The results revealed a significant difference between the priming and control groups in Impartial Beneficence scores, *t*(149) = 3.60, *p* < 0.001, Cohen’s *d* = 0.59. Specifically, the priming group (*M* = 3.34, *SD* = 0.99) scored significantly higher than the control group (*M* = 2.80, *SD* = 0.85).

#### 5.2.6. Moral Differential Mode

An independent-samples *t*-test was conducted to compare moral differential mode scores between the priming and control conditions. Levene’s test was significant, *F* = 3.99, *p* = 0.048, indicating that the homogeneity of variance assumption was not met; therefore, Welch’s *t*-test was reported. A significant difference emerged in moral differential mode scores between the priming and control groups, *t*(133.45) = −5.04, *p* < 0.001, Cohen’s *d* = −0.82. The priming group (*M* = 1.20, *SD* = 0.54) scored significantly lower than the control group (*M* = 1.58, *SD* = 0.38), indicating a reduced tendency toward moral differentiation.

#### 5.2.7. Mediating Model Testing

Using Model 4 of the PROCESS macro in SPSS 26.0 (Hayes, 2013), we tested a mediation model in which experimental condition was specified as the independent variable, impartial beneficence as the mediator, and moral differential mode as the dependent variable. Experimental condition was treated as a categorical variable and dummy-coded as 0 = control condition and 1 = IWAH-priming condition. Therefore, a positive coefficient for experimental condition indicated a higher score in the IWAH-priming condition than in the control condition. Age, gender, and instrumental harm were included as covariates, with gender entered as a categorical covariate and female participants used as the reference category.

**Figure 2:**
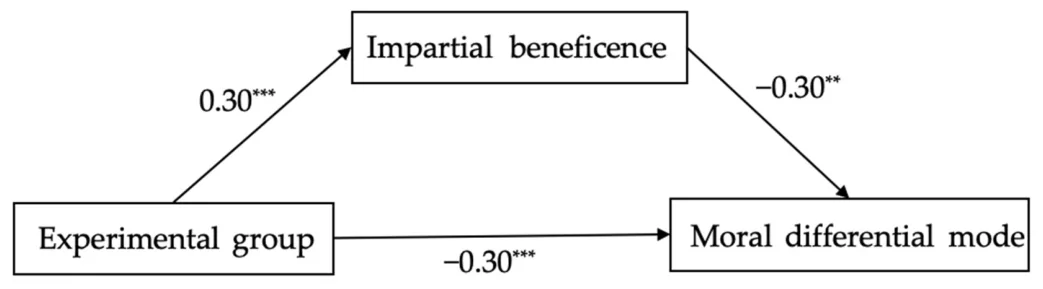
Mediation Model with Control Variables in Study 3. ** *p* < 0.01, *** *p* < 0.001.

The adjusted path coefficients, accounting for control variables, are presented in Figure 2, with detailed statistics provided in Table 10. Experimental condition significantly and positively predicted impartial beneficence (*B* = 0.57, *SE* = 0.12, *β* = 0.30, *t* = 4.62, *p* < 0.001) and significantly and negatively predicted moral differential mode (B = −0.30, *SE* = 0.08, *β* = −0.30, *t* = −3.84, *p* < 0.001). Impartial beneficence remained a significant negative predictor of moral differential mode (*B* = −0.16, *SE* = 0.05, *β* = −0.30, *t* = −3.20, *p* = 0.002). The bootstrapped 95% confidence interval for the indirect effect was [−0.18, −0.03], also excluding zero, thereby confirming the significance of the mediating effect.

Regarding control variables, gender had no significant effect (*p* > 0.512). Instrumental harm significantly predicted impartial beneficence (*B* = 0.47, *SE* = 0.06, *β* = 0.54, *t* = 8.22, *p* < 0.001) but did not significantly predict moral differential mode (*p* = 0.253). Age significantly predicted moral differential mode (*B* = 0.02, *SE* = 0.08, *β* = 0.17, *t* = 2.40, *p* = 0.017), but had no significant effect on impartial beneficence (*p* = 0.915). The corresponding direct and indirect effects are reported in Table 11.

### 5.3. Discussion

Study 3 used an experimental method to verify the causal relationship between IWAH and moral differential mode, and further verified the mediating role of impartial beneficence in this relationship.

Consistent with previous studies (Sparkman et al., 2022), viewing images of the Earth and related textual materials can significantly increase participants’ levels of IWAH. Furthermore, activating participants’ IWAH can significantly increase their tendency toward impartial beneficence and reduce moral differential mode. Consistent with H2, the results of the mediation model indicate that impartial beneficence partially mediates the relationship between IWAH and moral differential mode. Even after controlling for demographic variables and instrumental harm, the direct and mediating paths remain significant, demonstrating the robustness of the model.

The robust priming effects observed in Study 3 are particularly noteworthy, especially when compared to the relatively weaker IWAH priming effects often documented in Western samples (e.g., Reese & Kohlmann, 2015; Sparkman et al., 2022). This discrepancy may partially stem from the nature of the dependent variables. Previous Western studies frequently utilized specific, high-cost behavioral intentions (e.g., willingness to donate money or engage in climate action) as outcomes, where the abstract scale of “all humanity” can induce a sense of low collective efficacy or diffusion of responsibility (Markowitz & Shariff, 2012). In contrast, the current study utilized the moral differential mode—a cognitive representation and structural mapping of moral boundaries (Crimston et al., 2016). It appears that while a brief experimental prime may struggle to instantly mobilize costly behavioral sacrifices, it is highly effective at fundamentally reorganizing individuals’ underlying cognitive frameworks regarding who deserves moral consideration (Rottman et al., 2021).

Furthermore, this study found that activating IWAH significantly increases participants’ impartial beneficence orientation but does not affect their instrumental harm levels, further validating that impartial beneficence and instrumental harm are two relatively independent dimensions, supporting the utilitarian two-dimensional model (Kahane et al., 2018).

## 6. Study 4: The Influence of IWAH on Moral Differential Mode and the Mediating Role of Impartial Beneficence and the Moderating Role of Empathy

### 6.1. Method

Study 4 aims to investigate the moderating role of empathy in the relationship between IWAH and moral differential mode. This study adopts a survey-based approach to assess participants’ IWAH, impartial beneficence, moral differential mode, and empathy. In addition, it includes demographic variables and instrumental harm as covariates to examine how the four components of empathy differentially moderate the relationship between IWAH and moral differential mode.

#### 6.1.1. Participants

A total of 297 valid responses were collected, consisting of 164 males and 133 females, with an average age of 24.54 years (*SD* = 4.22). Participants were recruited through an online platform, and the questionnaire was distributed using the survey tool QuestionStar.

#### 6.1.2. Measures

The Chinese versions of the IWAH Scale, the Moral Expansiveness Scale, and the Oxford Utilitarianism Scale were used to measure identification with all humanity, moral circles, and impartial beneficence, respectively. All scales were identical to those employed in Study 3.

Empathy was assessed using the Chinese version of the Brief Interpersonal Reactivity Index (B-IRI; Ingoglia et al., 2016), which includes four subscales: Personal Distress, Empathic Concern, Perspective Taking, and Fantasy (see Appendix E). The Personal Distress subscale includes 4 items (e.g., “When I see someone who badly needs help in an emergency, I go to pieces”). The Empathic Concern subscale includes 4 items (e.g., “I would describe myself as a pretty soft-hearted person”), which contains one reverse-scored item (“When I see someone being treated unfairly, I sometimes don’t feel very much pity for them”). The Perspective Taking subscale consists of 4 items (e.g., “I sometimes try to understand my friends better by imagining how things look from their perspective”). The Fantasy subscale consists of 4 items (e.g., “After seeing a play or movie, I have felt as though I were one of the characters”). Participants rated each item on a 6-point Likert scale ranging from 1 (does not describe me well) to 6 (describes me very well). Mean scores were calculated for each subscale, with higher scores indicating stronger respective empathic traits. In the present study, the Cronbach’s *α* coefficient for the overall B-IRI was 0.746, and the *α* coefficients for the Personal Distress, Empathic Concern, Perspective Taking, and Fantasy subscales were 0.85, 0.58, 0.71, and 0.78, respectively.

Demographic Information. Demographic information such as gender and age was also collected.

### 6.2. Results

#### 6.2.1. Common Method Bias Test

Given that the data were collected via self-report questionnaires, we conducted an exploratory factor analysis to assess potential common method bias (Zhou & Long, 2004). According to Harman’s single-factor test, the first factor accounted for 15.03% of the total variance, which is well below the recommended threshold of 40%. Therefore, no substantial common method bias was identified in the current dataset.

#### 6.2.2. Confirmatory Factor Analysis

A confirmatory factor analysis tested the four-factor structure established in Study 1. The model showed acceptable overall fit, scaled *χ*^2^ (129) = 226.43, *p* < 0.001, robust CFI = 0.934, robust TLI = 0.922, robust RMSEA = 0.046, 90% CI [0.033, 0.058], and SRMR = 0.051. The standardized factor loadings ranged from 0.32 to 0.76 and were all statistically significant. These results provided support for applying the Study 1 target grouping in Study 4.

#### 6.2.3. Correlation Analysis

The results of the descriptive statistical analysis are presented in Table 12. IWAH was positively correlated with impartial beneficence (*r* = 0.21, *p* < 0.001), fantasy (*r* = 0.22, *p* < 0.001), perspective taking (*r* = 0.17, *p* = 0.046), and empathic concern (*r* = 0.29, *p* < 0.001), and negatively correlated with moral differential mode (*r* = −0.25, *p* < 0.001). Impartial beneficence showed a significant negative correlation with moral differential mode (*r* = −0.18, *p* = 0.002) and significant positive correlations with instrumental harm (*r* = 0.60, *p* < 0.001) and personal distress (*r* = 0.13, *p* = 0.024). Personal distress was strongly positively correlated with perspective taking (*r* = 0.75, *p* < 0.001) but negatively correlated with empathic concern (*r* = −0.17, *p* = 0.004). Fantasy was significantly positively correlated with both perspective taking (*r* = 0.31, *p* < 0.001) and empathic concern (*r* = 0.51, *p* < 0.001). Empathic concern was also positively correlated with perspective taking (*r* = 0.13, *p* = 0.023) and negatively correlated with moral differential mode (*r* = −0.15, *p* = 0.012). Regarding control variables, instrumental harm was negatively correlated with fantasy (*r* = −0.12, *p* = 0.034), and personal distress was positively correlated (*r* = 0.17, *p* = 0.004).

#### 6.2.4. Mediating Model Testing

Using Model 4 in the PROCESS macro (Hayes, 2013) for SPSS 26.0, we constructed a mediation model to examine the role of impartial beneficence in the relationship between IWAH and moral differential mode. IWAH served as the independent variable, moral differential mode as the dependent variable, and impartial beneficence as the mediator. Demographic variables and instrumental harm were included as covariates.

**Figure 3:**
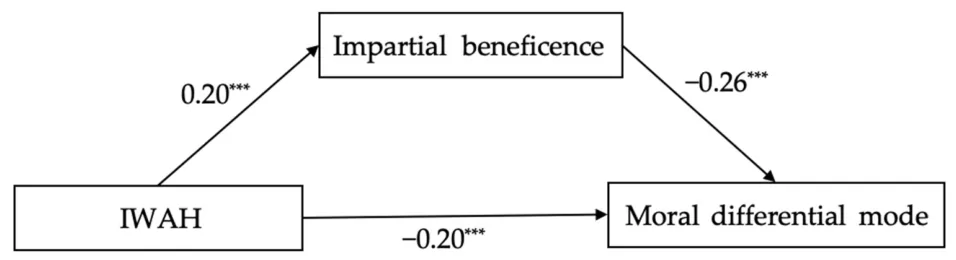
Mediation Model with Control Variables in Study 4. *** *p* < 0.001.

When covariates were included, the path coefficients are shown in Figure 3 and Table 13. IWAH significantly positively predicted impartial beneficence (*B* = 0.20, *SE* = 0.05, *β* = 0.20, *t* = 4.43, *p* < 0.001) and significantly negatively predicted moral differential mode (*B* = −0.11, *SE* = 0.03, *β* = −0.20, *t* = −3.50, *p* < 0.001). Moreover, impartial beneficence remained a significant negative predictor of moral differential mode (*B* = −0.14, *SE* = 0.04, *β* = −0.26, *t* = −3.63, *p* < 0.001). The Bootstrap analysis (10,000 samples) yielded a 95% confidence interval of [−0.10, −0.02], excluding zero, again confirming a significant mediating effect. Gender and age were not significant predictors (*ps* > 0.094). However, instrumental harm significantly predicted both impartial beneficence (*B* = 0.48, *SE* = 0.04, *β* = 0.60, *t* = 13.26, *p* < 0.001) and moral differential mode (*B* = 0.09, *SE* = 0.03, *β* = 0.21, *t* = 2.99, *p* = 0.003). The corresponding direct and indirect effects are presented in Table 14.

#### 6.2.5. Moderator Model Testing

Using Model 5 from the PROCESS macro in SPSS 26.0 (Hayes, 2013), four moderation models were constructed to examine the moderating roles of personal distress, empathic concern, perspective taking, and fantasy in the relationship between IWAH and moral differential mode, as well as the mediating role of impartial beneficence. Each model included IWAH as the independent variable, moral differential mode as the dependent variable, and impartial beneficence as the mediating variable. The four components of empathy were included as moderators, while demographic variables and instrumental harm were incorporated as control variables.

The results indicated that, regardless of whether control variables were included, only personal distress (without control variables: *B* = 0.06, *SE* = 0.02, *β* = 0.13, *t* = 2.53, *p* = 0.012; with control variables: *B* = 0.05, *SE* = 0.02, *β* = 0.11, *t* = 2.12, *p* = 0.035) and perspective taking (without control variables: *B* = 0.20, *SE* = 0.08, *β* = 0.13, *t* = 2.36, *p* = 0.018; with control variables: *B* = 0.18, *SE* = 0.08, *β* = 0.12, *t* = 2.08, *p* = 0.038) showed significant moderating effects. Fantasy (without control variables: *B* = −0.04, *β* = −0.06, *p* = 0.293; with control variables: *B* = −0.04, *β* = −0.05, *p* = 0.358) and empathic concern (without control variables: *B* = −0.03, *β* = −0.04, *p* = 0.438; with control variables: *B* = −0.02, *β* = −0.02, *p* = 0.628) did not exhibit significant effects.

(1) Moderation model including personal distress.

**Figure 4:**
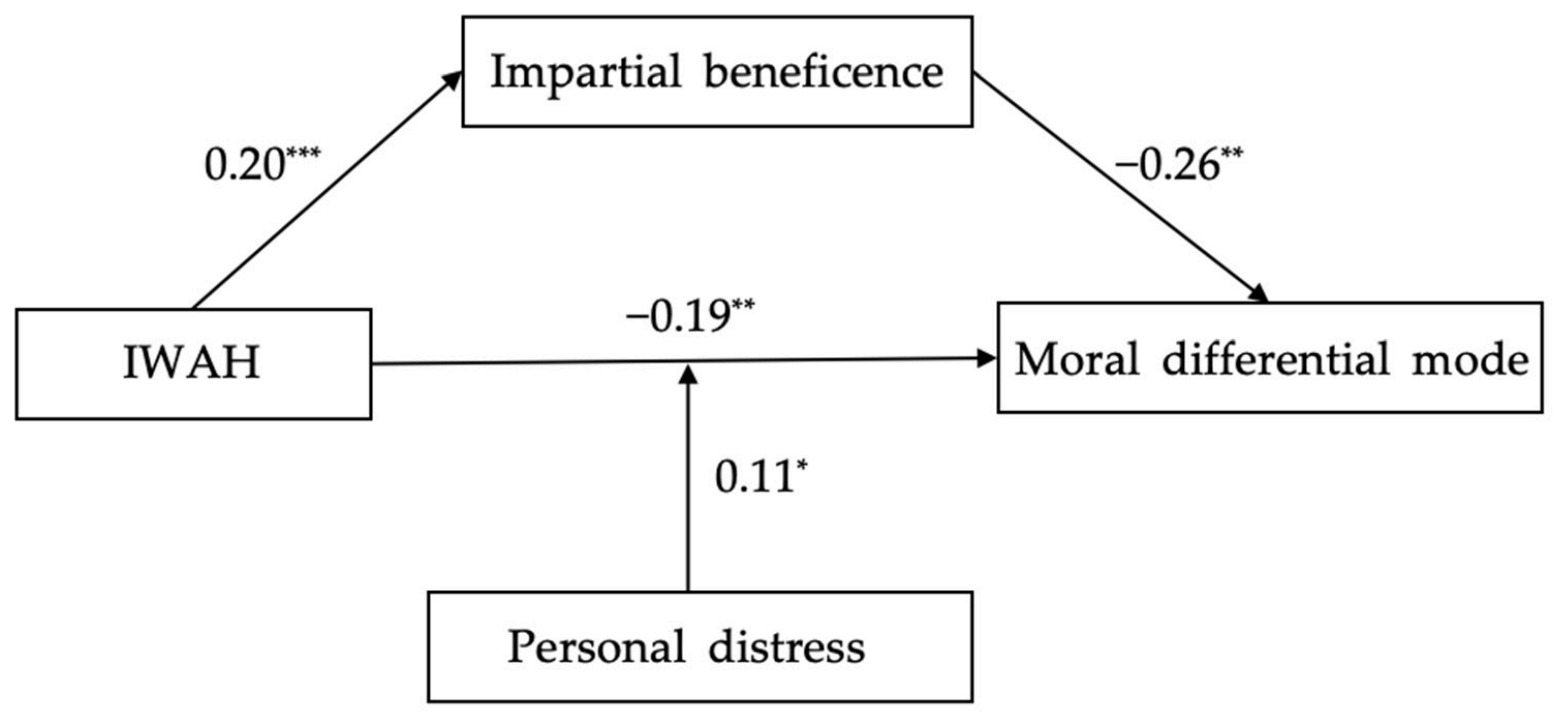
Moderating Model with Personal Distress (with Control Variables). * *p* < 0.05, ** *p* < 0.01, *** *p* < 0.001.

The path coefficients with control variables included are presented in Figure 4, with specific results shown in Table 15. IWAH significantly positively predicted impartial beneficence (*B* = 0.20, *SE* = 0.05, *β* = 0.20, *t* = 4.43, *p* < 0.001) and significantly negatively predicted moral differential mode (*B* = −0.28, *SE* = 0.09, *β* = −0.19, *t* = −3.16, *p* = 0.001). Impartial beneficence again significantly negatively predicted moral differential mode (*B* = −0.14, *SE* = 0.04, *β* = −0.26, *t* = −3.68, *p* < 0.001). Bootstrap analysis revealed a significant mediating effect, with the 95% confidence interval [−0.10, −0.02] excluding zero. The effect of personal distress on moral differential mode remained nonsignificant (*B* = −0.15, *β* = 0.08, *p* = 0.104), but the interaction term between IWAH and personal distress significantly predicted moral differential mode (*B* = 0.05, *SE* = 0.02, *β* = 0.11, *t* = 2.12, *p* = 0.035). Gender and age were not significant predictors (*ps* > 0.094), while instrumental harm significantly predicted both impartial beneficence (*B* = 0.48, *SE* = 0.04, *β* = 0.60, *t* = 13.27, *p* < 0.001) and moral differential mode (*B* = 0.08, *SE* = 0.03, *β* = 0.18, *t* = 2.50, *p* = 0.013).

A simple slopes analysis was conducted by categorizing IWAH and personal distress into high and low groups based on one standard deviation above and below the mean. The results indicated that under low personal distress, IWAH significantly negatively predicted moral differential mode (*β__simple_* = −0.33, *t* = −4.58, *p* < 0.001); however, under high personal distress, the prediction was not significant (*β__simple_* = −0.08, *t* = −0.97, *p* = 0.332). This pattern remained consistent after controlling for covariates: IWAH significantly negatively predicted moral differential mode under low personal distress (*β__simple_* = −0.30, *t* = −4.01, *p* < 0.001), but not under high personal distress (*β__simple_* = −0.08, *t* = −1.02, *p* = 0.308).

(2) Moderated Model with Perspective Taking

**Figure 5:**
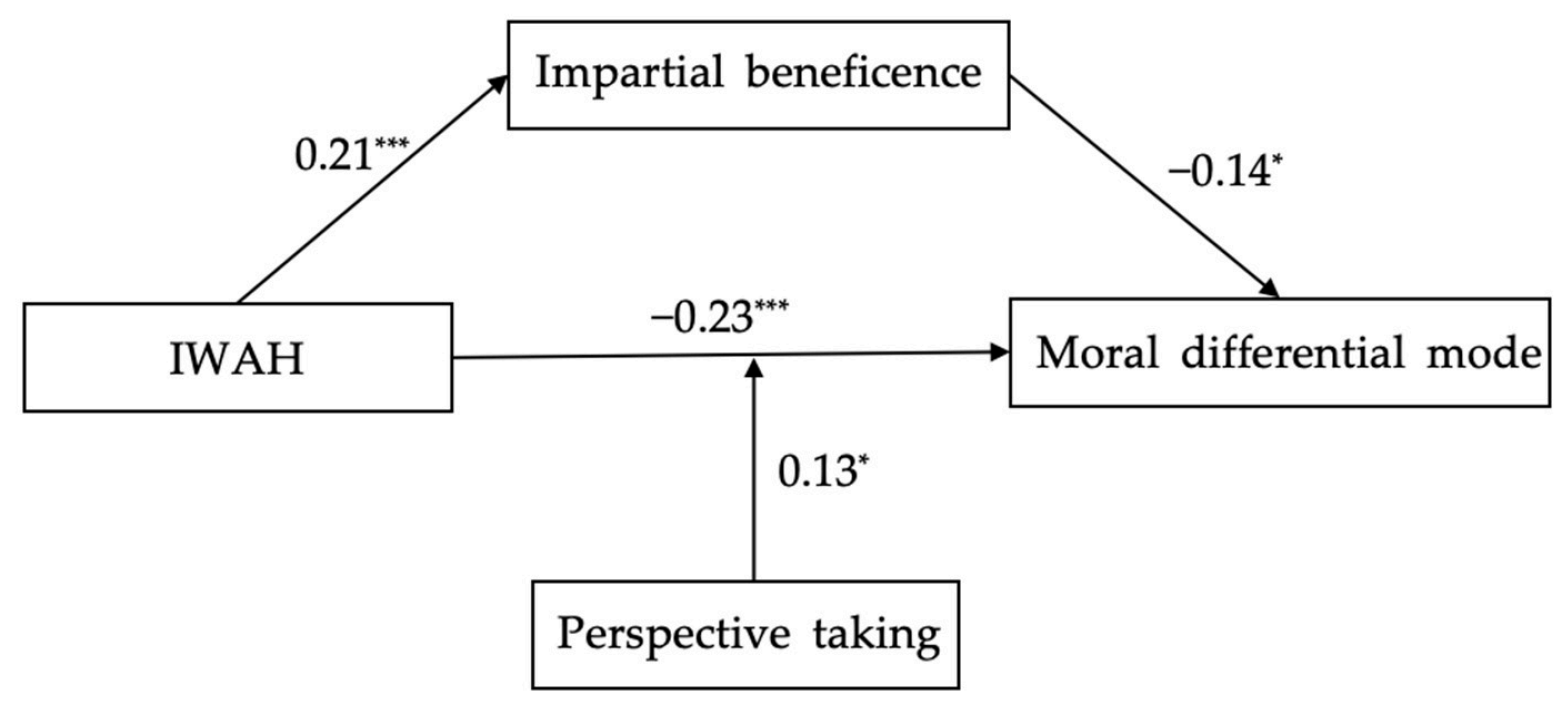
Moderating Model with Perspective Taking (with Control Variables). * *p* < 0.05, *** *p* < 0.001.

With control variables included, the path coefficient results are shown in Figure 5 and Table 16. IWAH remained a significant positive predictor of impartial beneficence (*B* = 0.20, *SE* = 0.05, *β* = 0.20, *t* = 4.43, *p* < 0.001) and a significant negative predictor of moral differential mode (*B* = −0.47, *SE* = 0.18, *β* = −0.20, *t* = −2.67, *p* < 0.001). Impartial beneficence continued to significantly and negatively predict moral differential mode (*B* = −0.14, *SE* = 0.04, *β* = −0.26, *t* = −3.64, *p* < 0.001). The mediating effect of impartial beneficence was again supported by Bootstrap analysis (95% CI [−0.10, −0.02]), which did not include zero. Perspective taking did not significantly predict moral differential mode (*B* = −0.55, *β* = 0.04, *p* = 0.464), but the interaction between IWAH and perspective taking remained significant (*B* = 0.18, *SE* = 0.08, *β* = 0.12, *t* = 2.08, *p* = 0.038). Gender and age had no significant effects (*ps* > 0.093), while instrumental harm significantly predicted both impartial beneficence (*B* = 0.48, *SE* = 0.04, *β* = 0.60, *t* = 13.27, *p* < 0.001) and moral differential mode (*B* = 0.08, *SE* = 0.03, *β* = 0.19, *t* = 2.76, *p* = 0.006).

Further analysis was conducted using the simple slope method. Participants were categorized into high and low groups based on one standard deviation above or below the mean of perspective taking. Under conditions of low perspective taking, IWAH significantly and negatively predicted moral differential mode (*β__simple_* = −0.36, *t* = −4.54, *p* < 0.001). However, under conditions of high perspective taking, the predictive effect was not significant (*β__simple_* = −0.10, *t* = −1.18, *p* = 0.239). This pattern persisted after controlling for covariates: under low perspective taking, IWAH significantly predicted lower moral differential mode (*β__simple_* = −0.20, *t* = −4.03, *p* < 0.001); under high perspective taking, the effect remained nonsignificant (*β__simple_* = −0.05, *t* = −1.10, *p* = 0.272).

### 6.3. Discussion

Study 4 further confirmed that IWAH significantly and negatively predicts moral differential mode, and that impartial beneficence mediates this relationship. Additionally, the study revealed that personal distress and perspective taking moderate the relationship between IWAH and moral differential mode, whereas empathic concern and fantasy do not exert significant moderating effects.

The results from the moderation model involving personal distress indicated that, although personal distress does not significantly predict moral differential mode on its own, its interaction with IWAH significantly predicts moral differential mode. This finding suggests that personal distress moderates the effect of IWAH on reducing moral differential mode. Consistent with Hypothesis 3, as levels of personal distress increase, the negative predictive effect of IWAH on moral differential mode diminishes. Specifically, among individuals with lower levels of personal distress, IWAH significantly and negatively predicts moral differential mode; however, among those with higher levels of personal distress, this effect is not significant.

Similarly, the moderation model incorporating perspective taking showed that perspective taking alone does not significantly predict moral differential mode, but its interaction with IWAH does significantly predict moral differential mode. This indicates that perspective taking moderates the impact of IWAH on moral differential mode. Consistent with Hypothesis 5, as levels of perspective taking increase, the negative predictive effect of IWAH on moral differential mode becomes weaker. Specifically, IWAH significantly and negatively predicts moral differential mode among individuals low in perspective taking, but this predictive effect becomes nonsignificant among individuals high in perspective taking.

## 7. General Discussion

### 7.1. The Role of IWAH in Reducing the Moral Differential Mode

Across four studies, participants showed a highly consistent descriptive hierarchy in moral concern, with mean concern decreasing from the Inner Circle to the Outer Circle, the Fringes, and Outside the Moral Boundary. The four-factor target organization identified in Study 1 received acceptable confirmatory support in Studies 2 and 4 but weaker support in Study 3. We therefore interpret the evidence as demonstrating a robust ordering of moral concern across layers and a broadly replicable, rather than perfectly invariant, target structure.

Drawing on moral expansion theory, the results demonstrate a clear hierarchical structure within moral circles among Chinese participants: family members and close friends are placed at the core; in-groups (e.g., fellow citizens) and respected Figures (e.g., superiors) occupy an intermediate circle of moral concern; stigmatized groups (e.g., homosexual individuals) and out-groups (e.g., foreigners) reside at the fringes of moral concern; while morally condemned individuals (e.g., murderers) are excluded entirely. On this basis, we define moral differential mode as the difference in moral concern between the Inner Circle and Fringes composite scores, offering a framework for assessing disparities in moral regard across social categories.

Across four studies, IWAH consistently emerged as a negative predictor of moral differential mode. Study 1 showed that IWAH enhanced moral concern for targets in the fringes of moral concern without diminishing concern for inner circle targets, thus reducing moral differential mode. This finding aligns with previous research on the effects of IWAH on perceptions of in-groups and out-groups (e.g., Grimalda et al., 2018) and contributes to the ongoing debate about whether moral concern is a limited resource (Neldner et al., 2018; Waytz et al., 2019; Rottman et al., 2021). The findings suggest that IWAH expands the overall capacity for moral concern rather than redistributing a fixed resource—increasing concern for distant groups while maintaining concern for close others.

In line with the common ingroup identity model (Gaertner & Dovidio, 2000), Study 3 demonstrated that identification with a superordinate group—namely, all humanity—can be effectively activated through visual and textual prompts. This reclassification mechanism integrates previously excluded out-groups into a new in-group category, thereby eliciting greater moral concern and moral standing, and ultimately reducing moral differential mode. Beyond situational activation, identification with all humanity can also be fostered through sustained interventions such as meditation practices (Loy & Reese, 2019) and structured empathy training (Brito-Pons et al., 2018). These approaches suggest promising pathways to cultivate a more inclusive moral circle structure.

In summary, this study confirms that IWAH significantly reduces moral differential mode, thereby enriching the literature on the prosocial and moral psychological effects of global identity. Furthermore, the findings offer empirical support for the psychological value of the concept of a “shared future for humanity.”

### 7.2. The Mediating Role of Impartial Beneficence

This study employed both questionnaire and experimental methods to examine the partial mediating role of impartial beneficence in the relationship between IWAH and moral differential mode. The results indicate that IWAH reduces moral differential mode by increasing individuals’ tendency to adopt an impartial beneficence orientation.

Specifically, IWAH significantly enhances individuals’ tendency toward impartial beneficence. When people identify with humanity as a unified whole, they become more concerned with maximizing the well-being of all individuals. From the perspective of social identity theory, defining oneself as a member of the broader human family leads individuals to adapt their psychological orientations and behaviors to uphold collective human interests (Turner, 1999). As a result, they shift their focus from prioritizing the welfare of close-knit groups to promoting the interests of humanity at large. Furthermore, an impartial beneficence orientation significantly negatively predicts moral differential mode. When individuals seek to treat members of various groups fairly and impartially, they are less likely to make sharp moral distinctions between these groups, thereby reducing disparities in moral regard. Mediation model analyses confirm that impartial beneficence partially mediates the relationship between IWAH and moral differential mode. IWAH encourages individuals to pursue moral concern for all in a more impartial and inclusive way, rather than focusing solely on proximate or familiar groups, thus contributing to a more equitable structure of moral concern.

Additionally, the findings support the utilitarian two-dimensional model proposed by Kahane et al. (2018). Across Studies 2 to 4, consistent with prior findings among Chinese participants (M. Chen et al., 2021) and Western philosophers (Kahane et al., 2018), impartial beneficence and instrumental harm were significantly positively correlated. This suggests a theoretical unity between the two constructs, as they are both central components of utilitarian thought. However, the results also indicate that these dimensions are psychologically distinct. For example, while activating IWAH significantly increased individuals’ impartial beneficence orientation, it had no impact on their endorsement of instrumental harm. Moreover, moral differential mode was strongly negatively associated with impartial beneficence, but showed little relationship with instrumental harm. These findings support the notion that impartial beneficence and instrumental harm represent two relatively independent psychological structures at the individual level (e.g., Capraro et al., 2019). Future research should avoid conflating the two and instead investigate their distinct antecedents and consequences more deeply.

Furthermore, the unusually strong effects of IWAH observed across our studies compared to the Western literature likely reflect fundamental cultural differences in self-construal. Individuals in individualistic cultures predominantly possess an independent self-construal, making it psychologically demanding to subsume one’s distinct identity into a massive, abstract global collective (Markus & Kitayama, 1991). Conversely, individuals in collectivistic cultures, such as China, are chronically oriented toward interdependent self-construals (Triandis, 1995). Because Chinese individuals are culturally practiced at contextualizing the self within expanding social networks and relational hierarchies (Fei, 2019; Yang, 2004), they may exhibit a higher cognitive readiness to assimilate and respond to a superordinate group identity prime, such as the human family (Gaertner & Dovidio, 2000). In other words, expanding the boundary of an already relational mindset is a more fluid cognitive transition than shifting from an individualistic baseline to a global-collective one. Additionally, the contemporary macro-cultural narrative in China emphasizing a “Community with a Shared Future for Mankind” may act as a chronic macro-level prime, creating a fertile sociocognitive environment that enhances participants’ situational receptivity to global identity cues (S. X. Chen et al., 2023; Chiu & Hong, 2007).

In conclusion, this study identifies impartial concern as a key psychological mechanism through which IWAH reduces moral differential mode. These findings deepen our understanding of how global identity shapes moral reasoning and offer a novel theoretical lens for understanding the positive psychological implications of the concept of a “shared future for humanity”.

### 7.3. The Moderating Role of Empathy

This study found that personal distress and perspective-taking moderate the relationship between IWAH and moral differential mode. As levels of personal distress or perspective-taking increase, the effect of IWAH on reducing moral differential mode diminishes.

First, personal distress was shown to moderate the association between IWAH and moral differential mode. Specifically, among individuals with lower levels of personal distress, IWAH significantly reduced moral differential mode. However, this effect became nonsignificant among those with higher levels of personal distress. The findings suggest that IWAH enhances individuals’ moral concern for stigmatized groups and outgroups, thereby reducing moral differential mode. Personal distress is conceptualized as a self-oriented distress experienced when confronted with the distress of others. According to Batson (1991), individuals who experience high personal distress may attempt to mitigate their discomfort by employing avoidance strategies, such as distancing themselves from those in need or disengaging from moral concern for marginalized groups. These avoidance tendencies can attenuate the effect of IWAH on moral differential mode.

In addition, perspective-taking was also found to moderate the relationship between IWAH and moral differential mode. For individuals with lower levels of perspective-taking, IWAH significantly reduced moral differential mode, whereas for those with higher levels, the effect became nonsignificant. One possible explanation is that individuals with high levels of perspective-taking tend to consider the complexity of moral situations more comprehensively (Hortensius et al., 2016), thereby better understanding the legitimacy of differential moral concern. Moreover, consistent with the negative consequences of perspective-taking described by Epley et al. (2006) and Vorauer et al. (2009), individuals may internalize prevailing cultural norms that endorse moral differential mode and assume that others share these views. This perceived normativity may lead them to maintain rather than challenge moral hierarchies, thereby reducing the impact of IWAH.

Finally, the study found no evidence that empathy and fantasy moderated the relationship between IWAH and moral differential mode. A plausible explanation is that these variables share strong conceptual overlap with IWAH, leading to reduced discriminant power in moderation analyses.

In summary, this study identifies personal distress and perspective-taking as important moderators in the relationship between IWAH and moral differential mode. These findings provide additional insight into the mechanisms through which empathy-related traits influence moral evaluations, and they offer empirical support for the nuanced roles of different empathy components in shaping moral judgment.

### 7.4. Research Implications

From a theoretical perspective, this study advances understanding of the moral-psychological benefits associated with IWAH. By integrating Western moral circle theory with China’s differential hierarchy theory, it introduces a quantitative approach to assessing moral differentiation, thereby offering a more systematic depiction of the moral-psychological structure among Chinese individuals. This facilitates the integration of Eastern and Western moral frameworks and supports cross-cultural collaboration in addressing global moral challenges. Additionally, the study reaffirms the validity of the two-dimensional model of utilitarianism, highlighting the distinct psychological role of impartial beneficence and enriching the literature on individual-level utilitarian thinking.

In terms of practical implications, IWAH represents a key psychological construct for advancing the “community of shared future for mankind” initiative within the field of psychology. The study identifies its positive moral-psychological outcomes, which may enhance China’s narrative positioning on the global stage and foster broader international consensus around the shared future framework. Moreover, these findings provide actionable guidance for moral education in the new era, contributing to the advancement of socialist moral development with Chinese characteristics. Ultimately, by demonstrating how global identification can expand traditional moral boundaries to foster inclusive social attitudes, this research highlights a vital psychological pathway for achieving human sustainable development. Such cultural adaptation is essential for mobilizing the cross-national cooperation required to address shared global challenges.

### 7.5. Limitations and Future Directions

First, the sample in this study was limited to Chinese participants. Although cross-cultural similarities and differences in moral psychology were observed during the research process, these phenomena were not explored in depth. Additionally, the sample in this study was relatively young in terms of age. Future research could conduct cross-cultural comparative studies to deeply examine the similarities and differences in moral psychology between Eastern and Western cultures. For example, one could explore whether Westerners’ moral care structures exhibit hierarchical characteristics and whether the influence of IWAH on moral differential mode has cross-cultural universality. Additionally, future research should adopt a broader sample, encompassing individuals of different ages, educational levels, and social classes, to enhance the representativeness and reliability of the research findings.

Second, this study used images and text to activate IWAH, which may raise issues regarding the temporary nature of the activation effect. Future research could design more systematic intervention programs, such as integrating the cultivation of IWAH into ideological and political education classrooms or social practice activities, and conducting pre- and post-tests and longitudinal studies to deeply examine the long-term effects of cultivating IWAH and its sustained influence on moral differential mode.

Finally, this study focused on moral differential mode regarding human entities and did not consider non-human entities. The view that moral care is a zero-sum resource suggests that increased moral concern for human entities may reduce moral concern for non-human entities such as animals and plants. Future research could explore this further. Additionally, future studies could further develop measurement methods for moral differential mode. For example, scales tailored to the Chinese cultural context could be developed, or the number and diversity of entity types could be expanded to more accurately and comprehensively capture this complex psychological phenomenon.

## Figures and Tables

**Table 1:** Factor Loadings of Moral Expansiveness Scale Items in Study 1.

MES Entity	Inner Circle of Moral Concern	Outer Circle of Moral Concern	Fringes of Moral Concern	Outside the Moral Boundary
15. Family member	**0.699**	−0.021	0.033	−0.109
2. Close friend	**0.452**	0.170	0.073	−0.007
17. Co-worker	0.159	**0.781**	−0.150	0.067
8. Partner/Spouse	0.128	**0.737**	−0.102	0.002
6. Chinese citizen	−0.060	**0.740**	0.017	−0.056
7. Soldier	−0.224	**0.733**	0.122	−0.098
9. Somebody from your neighborhood	0.098	**0.730**	−0.067	0.128
12. President of the People’s Republic of China	0.067	**0.676**	−0.036	0.059
16. Charity worker	−0.081	**0.625**	0.164	−0.163
18. Homosexual individual	0.087	−0.277	**0.806**	0.012
14. Somebody with different religious beliefs	0.066	0.201	**0.586**	−0.006
1. Foreign citizen	0.090	0.194	**0.578**	0.116
3. Individual with a mental illness	−0.101	0.200	**0.558**	−0.006
4. Refugee	−0.063	0.324	**0.500**	−0.062
5. Member of a different organization	0.020	0.357	**0.362**	0.067
10. Murderer	0.153	−0.084	0.078	**0.751**
11. Terrorist	−0.199	−0.056	0.050	**0.725**
13. Child molester	−0.116	0.125	−0.058	**0.632**

**Note:** Principal axis factoring with promax rotation was used. The factor correlations were as follows: Inner Circle–Outer Circle, *r* = 0.37; Inner Circle–Fringes, *r* = 0.21; Inner Circle–Outside the Moral Boundary, *r* = −0.10; Outer Circle–Fringes, *r* = 0.68; Outer Circle–Outside the Moral Boundary, *r* = 0.01; and Fringes–Outside the Moral Boundary, *r* = 0.10. The complete pattern matrix is reported without suppressing smaller or negative loadings. Boldface indicates that the item belongs to the corresponding circle.

**Table 2:** Descriptive Statistics and Correlations for Study 1 (*N* = 301).

Variable	*M*	*SD*	1	2	3	4	5	6
1 Age	25.27	6.26	-					
2 Inner Circle of Moral Concern	2.86	0.32	−0.08	-				
3 Outer Circle of Moral Concern	2.23	0.57	0.19 **	0.38 ***	-			
4 Fringes of Moral Concern	1.57	0.67	0.02	0.29 ***	0.69 ***	-		
5 Outside the Moral Boundary	0.18	0.41	0.09	−0.11	−0.01	0.09	-	
6 IWAH	3.67	0.91	0.03	0.04	0.33 ***	0.38 ***	−0.01	-
7 moral differential mode	1.29	0.65	−0.06	0.19 **	−0.53 ***	−0.89 ***	−0.14 *	−0.37 ***

* *p* < 0.05, ** *p* < 0.01, *** *p* < 0.001.

**Table 3:** Linear Regression Analysis of IWAH Predicting moral differential mode in Study 1 (*N* = 301).

Predictor Measures	Step 1			Step 2		
	*B*	*SE*	95%CI	*B*	*SE*	95%CI
Gender				−0.02	0.07	[−0.16, 0.12]
Age				−0.01	0.01	[−0.02, 0.01]
IWAH	−0.26 ***	0.04	[−0.34, −0.18]	−0.26 ***	0.04	[−0.34, −0.18]
*R* ^2^		0.14			0.14	
*F*		47.08 ***			15.96 ***	

*** *p* < 0.001.

**Table 4:** Linear Regression Analysis of IWAH Predicting Inner Circle of Moral Concern in Study 1 (*N* = 301).

Predictor Measures	Step 1			Step 2		
	*B*	*SE*	95%CI	*B*	*SE*	95%CI
Gender				−0.06	0.04	[−0.13, 0.02]
Age				−0.003	0.003	[−0.01, 0.002]
IWAH	0.02	0.04	[−0.02, 0.05]	0.01	0.02	[−0.03, 0.05]
*R* ^2^		0.002			0.17	
*F*		0.57			1.69	

**Table 5:** Linear Regression Analysis of IWAH Predicting Fringes of Moral Concern in Study 1 (*N* = 301).

Predictor Measures	Step 1			Step 2		
	*B*	*SE*	95%CI	*B*	*SE*	95%CI
Gender				−0.04	0.07	[−0.18, 0.10]
Age				0.002	0.006	[−0.01, 0.01]
IWAH	0.28 ***	0.04	[0.20, 0.36]	0.28 ***	0.04	[0.20, 0.36]
*R* ^2^		0.14			0.15	
*F*		50.43 ***			16.80 ***	

*** *p* < 0.001.

**Table 6:** Descriptive Statistics and Correlations for Study 2 (*N* = 303).

Variable	*M*	*SD*	1	2	3	4
1 Age	23.50	4.27	-			
2 Instrumental harm	3.08	1.24	0.16 **	-		
3 IWAH	3.75	0.95	−0.03	0.09	-	
4 Impartial beneficence	3.27	1.09	0.17 **	0.73 ***	0.21 ***	-
5 Moral differential mode	1.48	0.50	0.04	−0.11	−0.27 ***	−0.22 ***

** *p* < 0.01, *** *p* < 0.001.

**Table 7:** Mediation Model With Control Variables in Study 2 (*N* = 303).

	Impartial Beneficence			Moral Differential Mode		
	*B*	*SE*	*β*	*B*	*SE*	*β*
Control variable:						
Gender	0.06	0.08	0.06	−0.01	0.06	−0.01
Age	0.02	0.01	0.03	0.01	0.01	0.06
Instrumental harm	0.62 ***	0.03	0.71 ***	0.04	0.03	0.09
Predictor measures:						
IWAH	0.17 ***	0.04	0.15 ***	−0.12 ***	0.03	−0.22 ***
Mediating variable:						
Impartial beneficence				−0.11 **	0.04	−0.25 **
*R* ^2^	0.55			0.11		
*F*	93.60 ***			7.20 ***		

** *p* < 0.01, *** *p* < 0.001.

**Table 8:** Bootstrap Analysis and Effect Sizes for the Mediation Effects in Study 2.

	Effect Size	Proportion (%)	95%CI	
Without control variable				
Direct effect	−0.23	85	−0.34	−0.12
Indirect effect	−0.04	15	−0.07	−0.01
Total effect	−0.27			
With control variable				
Direct effect	−0.22	85	−0.34	−0.11
Indirect effect	−0.04	15	−0.08	−0.01
Total effect	−0.26			

**Table 9:** Descriptive Statistics and Correlations for Study 3 (*N* = 151).

Variable	*M*	*SD*	1	2		3
1 Age	23.40	3.58	-			
2 Instrumental harm	2.81	1.09	0.04	-		
3 Impartial beneficence	3.07	0.96	0.04	0.54 ***		-
4 Moral differential mode	1.39	0.51	0.16 *	−0.05	−0.38 ***	−0.32 ***

* *p* < 0.05, *** *p* < 0.001.

**Table 10:** Mediation Model With Control Variables in Study 3 (*N* = 151).

	Impartial Beneficence			Moral Differential Mode		
	*B*	*SE*	*β*	*B*	*SE*	*β*
Control variable:						
Gender	0.08	0.12	0.04	−0.05	0.08	−0.05
Age	0.001	0.02	0.01	0.02 *	0.01	0.18 *
Instrumental harm	0.47 ***	0.06	0.54 ***	0.05	0.04	0.10
Predictor measure:						
Experimental condition	0.57 ***	0.12	0.30 ***	−0.30 ***	0.08	−0.30 ***
Mediating variable:						
Impartial beneficence				−0.16 **	0.05	−0.30 **
*R* ^2^	0.38			0.24		
*F*	22.52 ***			8.94 ***		

* *p* < 0.05, ** *p* < 0.01, *** *p* < 0.001; Experimental condition (0 = control, 1 = IWAH priming).

**Table 11:** Bootstrap Analysis and Effect Sizes for the Mediation Effects in Study 3.

	Effect Size	Proportion (%)	95%CI	
Without Control variable				
Direct effect	−0.32	82	−0.47	−0.16
Indirect effect	−0.07	18	−0.14	−0.02
Total effect	−0.39			
With Control variable				
Direct effect	−0.30	77	−0.45	−0.14
Indirect effect	−0.09	23	−0.18	−0.03
Total effect	−0.39			

**Table 12:** Descriptive Statistics and Correlations for Study 4 (*N* = 297).

Variable	*M*	*SD*	1	2	3	4	5	6	7	8
1 Age	24.54	4.23	-							
2 Instrumental harm	2.92	1.12	−0.05	-						
3 IWAH	3.48	0.90	−0.10	0.02	-					
4 Impartial beneficence	3.12	0.91	0.03	0.60 ***	0.21 ***	-				
5 Personal distress	3.55	1.10	−0.07	0.17 **	−0.04	0.13 *	-			
6 Fantasy	4.74	0.73	−0.01	−0.12 *	0.22 ***	−0.03	0.07	-		
7 Perspective taking	2.07	0.35	−0.06	0.08	0.12 *	0.11	0.75 ***	0.31 ***	-	
8 Empathy concern	4.57	0.72	−0.06	−0.09	0.29 ***	0.04	−0.17 **	0.51 ***	0.13 *	-
9 Moral differential mode	1.56	0.48	−0.01	0.05	−0.25 ***	−0.18 **	0.09	−0.08	0.01	−0.15 *

* *p* < 0.05, ** *p* < 0.01, *** *p* < 0.001.

**Table 13:** Mediation Model With Control Variables in Study 4 (*N* = 297).

	Impartial Beneficence			Moral Differential Mode		
	*B*	*SE*	*β*	*B*	*SE*	*β*
Control variable:						
Gender	0.09	0.08	0.05	−0.02	0.05	−0.02
Age	0.02	0.01	0.07	−0.001	0.01	−0.02
Instrumental harm	0.48 ***	0.04	0.60 ***	0.09 **	0.03	0.21 **
Predictor measure:						
IWAH	0.20 ***	0.05	0.20 ***	−0.11 ***	0.03	−0.20 ***
Mediating variable:						
Impartial beneficence				−0.14 ***	0.04	−0.26 ***
*R* ^2^	0.41			0.11		
*F*	50.44 ***			7.10 ***		

** *p* < 0.01, *** *p* < 0.001.

**Table 14:** Bootstrap Analysis and Effect Sizes for the Mediation Effects in Study 4.

	Effect Size	Proportion (%)	95%CI	
Direct effect	−0.12	89	−0.18	−0.06
Indirect effect	−0.02	11	−0.03	−0.003
Total effect	−0.13			
With Control variable				
Direct effect	−0.11	79	−0.17	−0.05
Indirect effect	−0.03	21	−0.05	−0.01
Total effect	−0.13			

**Table 15:** Moderation Model With Personal Distress and With Control Variables in Study 4 (*N* = 297).

	Impartial Beneficence			Moral Differential Mode		
	*B*	*SE*	*β*	*B*	*SE*	*β*
Control variable:						
Gender	0.09	0.08	0.05	0.001	0.06	0.001
Age	0.02	0.01	0.08	−0.001	0.01	−0.005
Instrumental harm	0.48	0.04	0.60 ***	0.08	0.03	0.18 *
Predictor measure:						
IWAH	0.20 ***	0.05	0.20 ***	−0.28 **	0.09	−0.19 **
Personal distress				−0.15	0.09	0.08
IWAH × Personal distress				0.05 *	0.02	0.11 *
Mediating variable:						
Impartial beneficence				−0.14 ***	0.04	−0.26 ***
*R* ^2^	0.41			0.13		
*F*	50.44 ***			6.10 ***		

* *p* < 0.05, ** *p* < 0.01, *** *p* < 0.001.

**Table 16:** Moderation Model With Perspective Taking and With Control Variables in Study 4 (*N* = 297).

	Impartial Beneficence			Moral Differential Mode		
	*B*	*SE*	*β*	*B*	*SE*	*β*
Control variable:						
Gender	0.09	0.08	0.05	−0.01	0.06	−0.01
Age	0.02	0.01	0.08	−0.001	0.01	−0.004
Instrumental harm	0.48 ***	0.04	0.60 ***	0.08 **	0.03	0.19 **
Predictor measure:						
IWAH	0.20 ***	0.05	0.20 ***	−0.47 **	0.09	−0.20 ***
Perspective taking				−0.55	0.09	0.04
IWAH × Perspective taking				0.18 *	0.02	0.12 *
Mediating variable:						
Impartial beneficence				−0.14 ***	0.04	−0.26 ***
*R* ^2^	0.41			0.12		
*F*	50.44 ***			5.84 ***		

* *p* < 0.05, ** *p* < 0.01, *** *p* < 0.001.

## Data Availability

The original contributions presented in this study are included in the Supplementary Materials. Further inquiries can be directed to the corresponding author.

## Supplementary Materials

The following supporting information can be downloaded at https://www.mdpi.com/article/10.3390/bs16091541/s1. Supplementary Materials S1: Original datasets. Table S1: Additional demographic characteristics.

## Appendix A. Identification with All Humanity Scale (IWAH)

**Table A1:** Identification with All Humanity Scale (IWAH).

Item		1	2	3	4	5	6
1. How close do you feel to each of the following groups? (1 = not at all close, 6 = very close)	People in my community						
	People in my country						
	People anywhere in the world						
2. How often do you use the word “we” to refer to the following groups of people? (1 = almost never, 6 = very often)	People in my community						
	People in my country						
	People anywhere in the world						
3. How much would you say you have in common with the following groups? (1 = almost nothing in common, 6 = very much in common)	People in my community						
	People in my country						
	People anywhere in the world						
4. Sometimes people think of those who are not a part of their immediate family as “family.” To what degree do you think of the following groups of people as “family”? (1 = not at all, 6 = very much)	People in my community						
	People in my country						
	People anywhere in the world						
5. How much do you identify with (that is, feel a part of, feel love toward, have concern for) each of the following? (1 = not at all, 6 = very much)	People in my community						
	People in my country						
	People anywhere in the world						
6. How much would you say you care (feel upset, want to help) when bad things happen to: (1 = not at all, 6 = very much)	People in my community						
	People in my country						
	People anywhere in the world						
7. How much do you want to be: (1 = not at all, 6 = very much)	a. a responsible citizen of my community						
	b. a responsible Chinese citizen						
	c. a responsible citizen of the world						
8. How much do you believe in: (1 = not at all, 6 = very much)	a. being loyal to my community						
	b. being loyal to China						
	c. being loyal to all mankind						
9. When they are in need, how much do you want to help: (1 = not at all, 6 = very much)	People in my community						
	People in my country						
	People anywhere in the world						

## Appendix B. The Moral Expansiveness Scale (MES)

Instruction: Please carefully read the definitions of the following four different types of moral care, and determine which of these four types the following individuals belong to?

**Table A2:** The Moral Expansiveness Scale (MES).

Inner Circle of Moral Concern: These entities deserve the highest level of moral concern and standing. You have a moral obligation to ensure their welfare and feel a sense of personal responsibility for their treatment. Outer Circle of Moral Concern: These entities deserve moderate moral concern and standing. You are concerned about their moral treatment; however, your sense of obligation and personal responsibility is greatly reduced. Fringes of Moral Concern: These entities deserve minimal moral concern and standing, but you are not morally obligated or personally responsible for their moral treatment. Outside the Moral Boundary: These entities deserve no moral concern or standing. Feeling concern or personal responsibility for their moral treatment is extreme or nonsensical.				

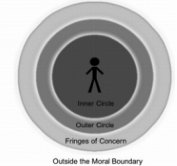

	Inner Circle of Moral Concern	Outer Circle of Moral Concern	Fringes of Moral Concern	Outside the Moral Boundary
1. Foreign citizen				
2. Close friend				
3. Individual with an intellectual disability				
4. Refugee				
5. Member of a different organization				
6. Chinese citizen				
7. Soldier				
8. Partner/Spouse				
9. Somebody from your neighborhood				
10. Murderer				
11. Terrorist				
12. The President of the People’s Republic of China (position not specific individual)				
13. Child molester				
14. Somebody with different religious beliefs				
15. Family member				
16. Charity worker				
17. Co-worker				
18. Homosexual individual				

## Appendix C. Oxford Utilitarianism Scale (OUS)

Instruction: Please read the following text carefully and rate the degree to which you agree or disagree with each statement. Please note that there is no right or wrong answer; simply answer based on your true thoughts (1 = strongly disagree, 6 = strongly agree).

**Table A3:** Oxford Utilitarianism Scale (OUS).

Item	1	2	3	4	5	6
1. From a moral perspective, people should not consider only the well-being of those who are especially close to them, but should care equally about the well-being of all people on Earth.						
2. From a moral point of view, we should feel obliged to give one of our kidneys to a person with kidney failure since we don’t need two kidneys to survive, but really only one to be healthy.						
3. If the only way to save another person’s life during an emergency is to sacrifice one’s own leg, then one is morally required to make this sacrifice.						
4. It is just as wrong to fail to help someone as it is to actively harm them yourself.						
5. It is morally wrong to keep money that one doesn’t really need if one can donate it to causes that provide effective help to those who will benefit a great deal.						
6. It is morally right to harm an innocent person if harming them is a necessary means to helping several other innocent people.						
7. If the only way to ensure the overall well-being and happiness of the people is through the use of political oppression for a short, limited period, then political oppression should be used.						
8. It is permissible to torture an innocent person if this would be necessary to provide information to prevent a bomb going off that would kill hundreds of people.						
9. Sometimes it is morally necessary for innocent people to die as collateral damage—if more people are saved overall.						

## Appendix D. Materials Used in Study3

**Table A4:** Materials Used in Study3.

Priming Condition
The United Nations Daily emphasizes that the world is undergoing profound changes unseen in a century, as global challenges such as climate change and public health crises continue to emerge. People around the world are increasingly aware that humanity shares one “global village” and together forms an interconnected community of shared destiny. Despite differences in history, culture, and levels of development, people across nations are all members of the human family, jointly pursuing peace, development, and other universal human values. It is essential to recognize that only through mutual care, solidarity, and cooperation among peoples worldwide can we collectively address global challenges, protect our common home, and work hand in hand to create a more prosperous and harmonious society.

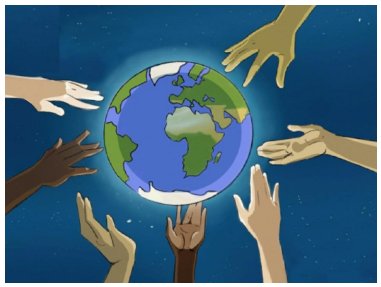

Do you agree with the viewpoints expressed in the text? How do you understand and uphold the idea of the human family?
Control Condition
The Art Review points out that white is one of the lightest colors and is commonly used in art. It is the color of snow, milk, and white paper, and can be produced by mixing the three primary colors in certain proportions. White objects completely reflect and scatter all visible wavelengths of light, often creating a sense of brightness and lightness. As a unique color, white plays an important role in artistic design. It is characterized by simplicity, brightness, and clarity, and is often used to convey minimalist and refined styles. At the same time, as an important means of creating artistic atmosphere, white provides infinite imaginative space, allowing viewers to freely associate it with countless images and meanings.

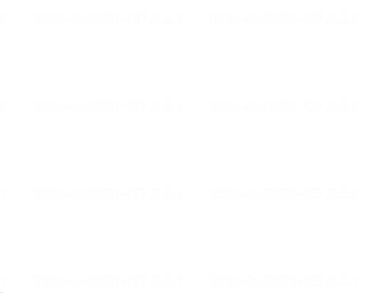

Do you agree with the viewpoints expressed in the text? How do you understand and make use of the color white?

## Appendix E. The Brief Interpersonal Reactivity Index (IRI)

Instruction: Please carefully read the following description and indicate how appropriate these sentences are in describing you (1 = Inappropriate, 6 = Very appropriate).

**Table A5:** The Brief Interpersonal Reactivity Index (IRI).

Item	1	2	3	4	5	6
1. I really get involved with the feelings of the characters in a novel.						
2. After seeing a play or movie, I have felt as though I were one of the characters.						
3. When I watch a good movie, I can very easily put myself in the place of a leading character.						
4. When I am reading an interesting story or novel, I imagine how I would feel if the events in the story were happening to me.						
5. I often have tender, concerned feelings for people less fortunate than me.						
6. When I see someone being taken advantage of, I feel kind of protective toward them.						
7. When I see someone being treated unfairly, I feel very much pity for them.						
8. I would describe myself as a pretty soft-hearted person.						
9. I try to look at everybody’s side of a disagreement before I make a decision.						
10. I sometimes try to understand my friends better by imagining how things look from their perspective.						
11. When I’m upset at someone, I usually try to “put myself in his shoes” for a while.						
12. Before criticizing somebody, I try to imagine how I would feel if I were in their place.						
13. In emergency situations, I feel apprehensive and ill-at-ease.						
14. Being in a tense emotional situation scares me.						
15. I tend to lose control during emergencies.						
16. When I see someone who badly needs help in an emergency, I go to pieces.						

## References

[B1-behavsci-16-01541] Abel L., Hornsey M., Hicks R. E. (2010). Social identity and moral judgement: The impact of political affiliation on the evaluation of government policy. Personality and individual differences: Current directions.

[B2-behavsci-16-01541] Aydın E., Bağci S. Ç., Keleşoğlu İ. (2022). Love for the globe but also the country matter for the environment: Links between nationalistic, patriotic, global identification and pro-environmentalism. Journal of Environmental Psychology.

[B3-behavsci-16-01541] Bassett J. F., Cleveland A. J. (2019). Identification with all humanity, support for refugees and for extreme counter-terrorism measures. Journal of Social and Political Psychology.

[B4-behavsci-16-01541] Batson C. D. (1991). The altruism question: Toward a social-psychological answer.

[B5-behavsci-16-01541] Batson C. D., Fultz J., Schoenrade P. A. (1987). Distress and empathy: Two qualitatively distinct vicarious emotions with different motivational consequences. Journal of Personality.

[B6-behavsci-16-01541] Bayram A. B. (2018). Nationalist cosmopolitanism: The psychology of cosmopolitanism, national identity, and going to war for the country. Nations and Nationalism.

[B7-behavsci-16-01541] Bloom P. (2010). How do morals change?. Nature.

[B8-behavsci-16-01541] Brito-Pons G., Campos D., Cebolla A. (2018). Implicit or explicit compassion? Effects of compassion cultivation training and comparison with mindfulness-based stress reduction. Mindfulness.

[B9-behavsci-16-01541] Capraro V., Everett J. A. C., Earp B. D. (2019). Priming intuition disfavors instrumental harm but not impartial beneficence. Journal of Experimental Social Psychology.

[B10-behavsci-16-01541] Chen M., Li W., Li X., Yuan B. (2021). Psychometric analysis of the Chinese version of the Oxford utilitarianism scale. Chinese Journal of Clinical Psychology.

[B11-behavsci-16-01541] Chen S. X., Ng J. C. K., Hui B. P. H., Au A. K. Y., Lam B. C. P., Wu W. C. H., Pun N., Beattie P., Welzel C., Liu J. H. (2023). Global consciousness predicts behavioral responses to the COVID-19 pandemic: Empirical evidence from 35 cultures. Social Psychological and Personality Science.

[B12-behavsci-16-01541] Chiu C. Y., Hong Y. Y., Kruglanski A. W., Higgins E. T. (2007). Cultural processes: Basic principles. Social psychology: Handbook of basic principles.

[B13-behavsci-16-01541] Crimston D., Bain P. G., Hornsey M. J., Bastian B. (2016). Moral expansiveness: Examining variability in the extension of the moral world. Journal of Personality and Social Psychology.

[B14-behavsci-16-01541] Crimston D., Hornsey M. J., Bain P. G., Bastian B. (2018). Toward a psychology of moral expansiveness. Current Directions in Psychological Science.

[B15-behavsci-16-01541] Deng X. (2021). Identification with all humanity and willingness to help people in COVID-19 affected countries: Testing a moderated mediation model. Personality and Individual Differences.

[B16-behavsci-16-01541] Epley N., Caruso E. M., Bazerman M. H. (2006). When perspective taking increases taking: Reactive egoism in social interaction. Journal of Personality and Social Psychology.

[B17-behavsci-16-01541] Fei X. (2019). From the soil: The foundations of Chinese society.

[B18-behavsci-16-01541] Gaertner S. L., Dovidio J. F. (2000). Reducing intergroup bias: The common ingroup identity model.

[B19-behavsci-16-01541] Galinsky A. D., Moskowitz G. B. (2000). Perspective-taking: Decreasing stereotype expression, stereotype accessibility, and in-group favoritism. Journal of Personality and Social Psychology.

[B20-behavsci-16-01541] Grimalda G., Buchan N., Brewer M. (2018). Social identity mediates the positive effect of globalization on individual cooperation: Results from international experiments. PLoS ONE.

[B21-behavsci-16-01541] Hamer K., Gutowski J., Scuzzarello S., Kinnvall C., Monroe K. R. (2009). Social identifications and pro-social activity in Poland. On behalf of others: The psychology of care in a global world.

[B22-behavsci-16-01541] Hamer K., Penczek M., Bilewicz M. (2017). “Humanum ignoscere est”. The relationship of national and supranational identifications with intergroup forgiveness. Personality and Individual Differences.

[B23-behavsci-16-01541] Hamer K., Penczek M., Bilewicz M. (2018). Between universalistic and defensive forms of group attachment. The indirect effects of national identification on intergroup forgiveness. Personality and Individual Differences.

[B24-behavsci-16-01541] Hayes A. F. (2013). Introduction to mediation, moderation, and conditional process analysis: A regression-based approach.

[B25-behavsci-16-01541] Hortensius R., Schutter D. J. L. G., Gelder B. (2016). Personal distress and the influence of bystanders on responding to an emergency. Cognitive, Affective & Behavioral Neuroscience.

[B26-behavsci-16-01541] Huang Z., Jing Y., Yu F., Gu R., Zhou X., Zhang J., Cai H. (2018). The rise of individualism and the decline of collectivism?—Global cultural change and psychological shifts. Advances in Psychological Science.

[B27-behavsci-16-01541] Ingoglia S., Lo Coco A., Albiero P. (2016). Development of a brief form of the interpersonal reactivity index (B–IRI). Journal of Personality Assessment.

[B28-behavsci-16-01541] Kahane G., Everett J. A. C., Earp B. D., Caviola L., Faber N. S., Crockett M. J., Savulescu J. (2018). Beyond sacrificial harm: A two-dimensional model of utilitarian psychology. Psychological Review.

[B29-behavsci-16-01541] Laham S. M. (2009). Expanding the moral circle: Inclusion and exclusion mindsets and the circle of moral regard. Journal of Experimental Social Psychology.

[B30-behavsci-16-01541] Loy L. S., Reese G. (2019). Hype and hope? Mind-body practice predicts pro-environmental engagement through global identity. Journal of Environmental Psychology.

[B31-behavsci-16-01541] Markowitz E. M., Shariff A. F. (2012). Climate change and moral judgement. Nature Climate Change.

[B32-behavsci-16-01541] Markus H. R., Kitayama S. (1991). Culture and the self: Implications for cognition, emotion, and motivation. Psychological Review.

[B33-behavsci-16-01541] McFarland S., Brown D. (2008). Who believes that identification with all humanity is ethical?. Psicología Política.

[B34-behavsci-16-01541] McFarland S., Hornsby W. (2015). An analysis of five measures of global human identification. European Journal of Social Psychology.

[B35-behavsci-16-01541] McFarland S., Webb M., Brown D. (2012). All humanity is my ingroup: A measure and studies of identification with all humanity. Journal of Personality and Social Psychology.

[B36-behavsci-16-01541] Neldner K., Crimston C., Wilks M., Redshaw J., Nielsen M. (2018). The developmental origins of moral concern: An examination of moral boundary decision making throughout childhood. PLoS ONE.

[B37-behavsci-16-01541] Paciello M., Fida R., Cerniglia L., Tramontano C., Cole E. (2013). High cost helping scenario: The role of empathy, prosocial reasoning and moral disengagement on helping behavior. Personality and Individual Differences.

[B38-behavsci-16-01541] Pinker S. (2011). The angels of our better nature: Why violence has declined.

[B39-behavsci-16-01541] Pong V., Tam K. P. (2023). Relationship between global identity and pro-environmental behavior and environmental concern: A systematic review. Frontiers in Psychology.

[B40-behavsci-16-01541] Reese G., Kohlmann F. (2015). Feeling global, acting ethically: Global identification and fairtrade consumption. The Journal of Social Psychology.

[B41-behavsci-16-01541] Rottman J., Crimston C. R., Syropoulos S. (2021). Tree-huggers versus human-lovers: Anthropomorphism and dehumanization predict valuing nature over outgroups. Cognitive Science.

[B42-behavsci-16-01541] Singer P. (1981). The expanding circle.

[B43-behavsci-16-01541] Sparkman D. J. (2023). Identification with humanity and health-related behaviors during COVID-19. Group Processes & Intergroup Relations.

[B44-behavsci-16-01541] Sparkman D. J., Kleive K., Ngu E. (2022). Does activating the human identity improve health-related behaviors during COVID-19?: A social identity approach. Frontiers in Psychology.

[B45-behavsci-16-01541] Triandis H. C. (1995). Individualism & collectivism.

[B46-behavsci-16-01541] Turner J. C., Ellemers N., Spears R., Doosje B. (1999). Some current issues in research on social identity and self-categorization theories. Social identity: Context, commitment, content.

[B47-behavsci-16-01541] Vorauer J. D., Martens V., Sasaki S. J. (2009). When trying to understand detracts from trying to behave: Effects of perspective taking in intergroup interaction. Journal of Personality and Social Psychology.

[B48-behavsci-16-01541] Waytz A., Iyer R., Young L., Haidt J., Graham J. (2019). Ideological differences in the expanse of the moral circle. Nature Communications.

[B49-behavsci-16-01541] Xu Y., Hamamura T. (2014). Folk beliefs of cultural changes in China. Frontiers in Psychology.

[B50-behavsci-16-01541] Yang G. (2004). The psychology and behavior of Chinese people: An indigenous study.

[B51-behavsci-16-01541] Yu F., Xu L. (2018). The moral structure of Chinese people: The differential moral circles. Journal of Nanjing Normal University: Social Science Edition.

[B52-behavsci-16-01541] Zeng Z. (2020). Confucian benevolence and the community with a shared future for mankind. Guangming Daily.

[B53-behavsci-16-01541] Zhou H., Long L. (2004). Statistical testing and control methods for common method bias. Advances in Psychological Science.

