# Supplementary material for: People Who Identify with All of Humanity Make Less of a Moral Distinction Between Close and Distant People
Source: Behav Sci (Basel). 2026 Sep 1;16(9):1541. doi: 10.3390/bs16091541 (PMC13603700; doi:10.3390/bs16091541)
Supplement: Supplementary file 1 [file behavsci-16-01541-s001.zip › Table S1 Additional demographic characteristics.pdf]

Table S1 Additional demographic characteristics

Variable	Category	N	%
**Education Level**	Junior high school and below	0	0.00%
	High school / Vocational school	6	1.98%
	Associate degree / Junior college	20	6.60%
	Bachelor's degree	255	84.16%
	Master's degree and above	22	7.26%
Monthly Income (RMB)	500 and below	19	6.27%
	501 – 2,000	102	33.66%
	2,001 – 4,000	56	18.48%
	4,001 – 6,000	83	27.39%
	6,001 – 8,000	31	10.23%
	8,001 – 10,000	10	3.30%
	10,001 – 15,000	0	0.00%
	15,001 – 20,000	0	0.00%
	20,001 – 30,000	1	0.33%
	Above 30,000	1	0.33%
Subjective SES	1 – Lowest social status	2	0.66%
	2	10	3.30%
	3	29	9.57%
	4	52	17.16%
	5	92	30.36%
	6	69	22.77%
	7	37	12.21%
	8	10	3.30%
	9	2	0.66%
	10 – Highest social status	0	0.00%
